# AI-guided discovery of the invariant host response to viral pandemics

**DOI:** 10.1016/j.ebiom.2021.103390

**Published:** 2021-06-11

**Authors:** Debashis Sahoo, Gajanan D. Katkar, Soni Khandelwal, Mahdi Behroozikhah, Amanraj Claire, Vanessa Castillo, Courtney Tindle, MacKenzie Fuller, Sahar Taheri, Thomas F. Rogers, Nathan Beutler, Sydney I. Ramirez, Stephen A. Rawlings, Victor Pretorius, Davey M. Smith, Dennis R. Burton, Laura E. Crotty Alexander, Jason Duran, Shane Crotty, Jennifer M. Dan, Soumita Das, Pradipta Ghosh

**Affiliations:** a Department of Pediatrics, University of California San Diego, 9500 Gilman Drive, MC 0730, Leichtag Building 132, La Jolla, CA 92093-0831, USA; b Department of Computer Science and Engineering, Jacobs School of Engineering, University of California San Diego, USA; c Moores Cancer Center, University of California San Diego, USA; d Department of Cellular and Molecular Medicine, University of California San Diego, USA; e Department of Immunology and Microbiology, The Scripps Research Institute, La Jolla, CA 92037, USA; f Division of Infectious Diseases, Department of Medicine, University of California, San Diego, La Jolla, CA 92037, USA; g IAVI Neutralizing Antibody Center, The Scripps Research Institute, La Jolla, CA 92037, USA; h Consortium for HIV/AIDS Vaccine Development (CHAVD), The Scripps Research Institute, La Jolla, CA 92037, USA; i Pulmonary Critical Care Section, Veterans Affairs (VA) San Diego Healthcare System, La Jolla, California; Division of Pulmonary, Critical Care and Sleep Medicine, Department of Medicine, University of California San Diego (UCSD), La Jolla, CA, USA; j Center for Infectious Disease and Vaccine Research, La Jolla Institute for Immunology (LJI), La Jolla, CA, USA; k Department of Medicine, Division of Infectious Diseases and Global Public Health, University of California, San Diego (UCSD), La Jolla, CA, USA; l Department of Pathology, University of California San Diego, USA; m Medicine, University of California San Diego, USA; n Department of Surgery, University of California San Diego, USA; o Division of Cardiology, Department of Internal Medicine, UC San Diego Medical Center, La Jolla 92037

**Keywords:** Artificial intelligence/machine learning, Boolean equivalent clusters, Angiotensin converting enzyme (ACE)-2, Coronavirus COVID-19, Immune response, Lung alveoli, Natural Killer (NK) cells, Interleukin 15 (IL15)

## Abstract

**Background:**

Coronavirus Disease 2019 (Covid-19) continues to challenge the limits of our knowledge and our healthcare system. Here we sought to define the host immune response, a.k.a, the “cytokine storm” that has been implicated in fatal COVID-19 using an AI-based approach.

**Method:**

Over 45,000 transcriptomic datasets of viral pandemics were analyzed to extract a 166-gene signature using ACE2 as a ‘seed’ gene; ACE2 was rationalized because it encodes the receptor that facilitates the entry of SARS-CoV-2 (the virus that causes COVID-19) into host cells. An AI-based approach was used to explore the utility of the signature in navigating the uncharted territory of Covid-19, setting therapeutic goals, and finding therapeutic solutions.

**Findings:**

The 166-gene signature was surprisingly conserved across all *vi*ral *p*andemics, including COVID-19, and a subset of 20-genes classified disease severity, inspiring the nomenclatures *ViP* and *severe-ViP* signatures, respectively. The *ViP* signatures pinpointed a paradoxical phenomenon wherein lung epithelial and myeloid cells mount an IL15 cytokine storm, and epithelial and NK cell senescence and apoptosis determine severity/fatality. Precise therapeutic goals could be formulated; these goals were met in high-dose SARS-CoV-2-challenged hamsters using either neutralizing antibodies that abrogate SARS-CoV-2•ACE2 engagement or a directly acting antiviral agent, EIDD-2801. IL15/IL15RA were elevated in the lungs of patients with fatal disease, and plasma levels of the cytokine prognosticated disease severity.

**Interpretation:**

The *ViP* signatures provide a quantitative and qualitative framework for titrating the immune response in viral pandemics and may serve as a powerful unbiased tool to rapidly assess disease severity and vet candidate drugs.

**Funding:**

This work was supported by the National Institutes for Health (NIH) [grants CA151673 and GM138385 (to DS) and AI141630 (to P.G), DK107585–05S1 (SD) and AI155696 (to P.G, D.S and S.D), U19-AI142742 (to S.C, CCHI: Cooperative Centers for Human Immunology)]; Research Grants Program Office (RGPO) from the University of California Office of the President (UCOP) (R00RG2628 & R00RG2642 to P.G, D.S and S.D); the UC San Diego Sanford Stem Cell Clinical Center (to P.G, D.S and S.D); LJI Institutional Funds (to S.C); the VA San Diego Healthcare System Institutional funds (to L.C.A). GDK was supported through The American Association of Immunologists Intersect Fellowship Program for Computational Scientists and Immunologists.

**One sentence summary:**

The host immune response in COVID-19.

**Panel: research in context**

**Evidence before this study**

The SARS-CoV-2 pandemic has inspired many groups to find innovative methodologies that can help us understand the host immune response to the virus; unchecked proportions of such immune response have been implicated in fatality. We searched GEO and ArrayExpress that provided many publicly available gene expression data that objectively measure the host immune response in diverse conditions. However, challenges remain in identifying a set of host response events that are common to every condition. There are no studies that provide a reproducible assessment of prognosticators of disease severity, the host response, and therapeutic goals. Consequently, therapeutic trials for COVID-19 have seen many more ‘misses’ than ‘hits’. This work used multiple (> 45,000) gene expression datasets from GEO and ArrayExpress and analyzed them using an unbiased computational approach that relies upon fundamentals of gene expression patterns and mathematical precision when assessing them.

**Added value of this study**

This work identifies a signature that is surprisingly conserved in all viral pandemics, including Covid-19, inspiring the nomenclature *ViP*-signature. All COVID-19 datasets were prospectively analyzed using the signatures, underscoring the utility of these signatures to navigate future pandemics. A subset of 20-genes classified disease severity in respiratory pandemics. The *ViP* signatures pinpointed the nature and source of the ‘cytokine storm’ mounted by the host. They also helped formulate precise therapeutic goals and rationalized the repurposing of FDA-approved drugs.

**Implications of all the available evidence**

The *ViP* signatures provide a quantitative and qualitative framework for assessing the immune response in emergent new diseases, such as the next viral pandemic; they serve as a powerful unbiased tool to rapidly define the disease, interrogate mechanisms, assess severity, set therapeutic goals and vet candidate drugs.

Alt-text: Unlabelled box

## 1 Introduction

As the rapidly unfolding COVID-19 pandemic claims its victims around the world, it has also inspired the scientific community to come up with solutions that have the potential to save lives. In the works are numerous investigational drugs at various phases of clinical trials, from rationalizing [1], to IRB approvals, recruitment and execution [2,3], all directed to meet an urgent and unmet need —i.e., ameliorate the severity of COVID-19 and reduce mortality.

Two obstacles make that task difficult—First, the pathophysiology of COVID-19 remains a mystery. The emerging reports generally agree that the disease has a very slow onset [4,5] and that those who succumb typically mount a ‘cytokine storm’ [4,6], i.e., an overzealous immune response. Despite being implicated as a cause of mortality and morbidity in COVID-19, we know virtually nothing about what constitutes (nature, extent) or contributes to (cell or origin) such an overzealous response. Consequently, treatment goals in COVID-19 have been formulated largely as a ‘trial and error’-approach; this is reflected in the mixed results of the trials that have concluded [7]. Second, there is no established pre-clinical animal or human cell/organoid models for COVID-19; vetting the accuracy and/or the relevance of such models requires first an understanding of the host response in the disease.

We set out to define this aberrant host immune response in COVID-19 using machine learning tools that can look beyond interindividual variability to extract underlying gene expression patterns within multidimensional complex data. The approach was used across multiple cohorts of viral pandemics. The resultant pattern, i.e., signature, was subsequently exploited as a predictive model to navigate COVID-19. Findings not only pinpointed the precise nature of the cytokine storm, the culprit cell types and the organs, but also revealed disease pathophysiology, and helped formulate specific therapeutic goals for reducing disease severity. Key findings were validated in preclinical models of COVID-19 in Syrian hamsters and in the lungs and plasma of infected patients.

## 2 Methods

### 2.1 Key resource table

MATERIALS & REAGENTS

**Table:** 

ANTIBODIES USED FOR IMMUNOCYTOCHEMISTRY
Name
**Manufacturer**
**Catalog number**
**Dilution factor**
IL15 (E-4)
Santa Cruz
sc-8437
1:10
IL15RA
Proteintech
16,744–1-AP
1:200
Goat anti-rabbit
Vector Laboratories, Burlingame, USA
MP-7401
1:500
Goat anti-mouse
Vector Laboratories, Burlingame, USA
MP-7402
1:500
INSTRUMENTS
Leica DMI4000B (Automated Inverted Microscope)
Leica Microsystems
DMI4000B
Power Pressure Cooker XL
Tristar Products
Fisherbrand™ 150 Handheld Homogenizer
Fisher Scientific
15,340,168
SOFTWARE
ImageJ
https://imagej.nih.gov/ij/index.html
GraphPad Prism
https://www.graphpad.com/scientific-software/prism/
KITS, ENZYMES, CHEMICALS, AND REAGENTS
ELISA MAX Deluxe set
Biolegend
435,104
V-PLEX sandwich immunoassays
Mesoscale Discovery (MSD)
K151A9H-1
Zinc Formalin
Fisher Scientific
23–313,096
Xylene
VWR
XX0060–4
Hematoxylin
Sigma-Aldrich Inc
MHS1
Ethanol
Koptec
UN1170
Sodium Citrate
Sigma-Aldrich
W302600
DAB (10X)
Vector Laboratories, Burlingame, USA
SK-4105
Hematoxylin
Sigma-Aldrich Inc. MO, USA
MHS1
Stable Peroxidase substrate buffer (10x)
Thermo Fisher
34,062
1:10
3% Hydrogen Peroxide
Target
245–07–3628
Horse Serum
Vector Labs
30,022
Paraformaldehyde 16% Solution, EM Grade
Electron Microscopy Sciences
15,710
100% Methanol
Supelco
MX0485
Glycine
Fisher Scientific
BP381–5
Bovine Serum Albumin
Sigma-Aldrich
A9647–100G
Triton-X 100
Sigma-Aldrich
X100–500ML
Prolong Glass
Invitrogen
P36984
Nail Polish (Rapid Dry)
Electron Microscopy Sciences
72,180
Gill Modified Hematoxylin (Solution II)
Millipore Sigma
65,066–85
Goat serum
Vector Laboratories
MP-7401
Quick-RNA MicroPrep Kit
Zymo Research
R1051
Quick-RNA MiniPrep Kit
Zymo Research
R1054
Ethyl alcohol, pure
Sigma-Aldrich
E7023
qScript cDNA SuperMix
Quanta Biosciences
95,048
OTHER
RNase Away
Thermo Fisher Scientific
14–375–35
Noyes Spring Scissors - Angled
Fine Science Tools
15,013–12

## 3 Reagent validation

There are no cell lines used in this work. Both antibodies (IL15 and IL15RA) used in this work have been previously validated for use in IHC studies. ELISA kits were validated using internal controls (standard curve).

## 4 Detailed methods

### 4.1 Data collection and annotation

Publicly available microarray and RNASeq databases were downloaded from the National Center for Biotechnology Information (NCBI) Gene Expression Omnibus (GEO) website [8], [9], [10]. Gene expression summarization was performed by normalizing Affymetrix platforms by RMA (Robust Multichip Average) [11,12] and RNASeq platforms by computing TPM (Transcripts Per Millions) [13] values whenever normalized data were not available in GEO. We used log2(TPM) if TPM > 1 and (TPM – 1) if TPM < 1 as the final gene expression value for analyses. A catalog of all datasets analyzed in this work can be found in **Supplementary Table 1**.

### 4.2 Rapid autopsy procedure for tissue collection

The lung specimens from the COVID 19 positive human subjects were collected using autopsy (study was IRB Exempt). All donations to this trial were obtained after telephone consent followed by written email confirmation with next of kin/power of attorney per California state law (no in-person visitation could be allowed into our COVID-19 ICU during the pandemic).

The team member followed the CDC guidelines for COVID19 and the autopsy procedures [8,9]. Lung specimens were collected in 10% Zinc-formalin and stored for 72 h before processing for histology. Patient characteristic is listed in **Supplementary Table 6**.

Autopsy #2 was a standard autopsy performed by anatomical pathology in the BSL3 autopsy suite. The patient expired and his family consented for autopsy. After 48 h, lungs were removed and immersion fixed whole in 10% formalin for 48 h and then processed further. Lungs were only partially fixed at this time (about 50% fixed in thicker segments) and were sectioned further into small 2–4 cm chunks and immersed in 10% formalin for further investigation.

Autopsies #4 and #5 were collected from rapid postmortem lung biopsies. The procedure was performed in the Jacobs Medical Center ICU (all of the ICU rooms have a pressure-negative environment, with air exhausted through HEPA filters [Biosafety Level 3 (BSL3)] for isolation of SARS-CoV-2 virus). Biopsies were performed 2–4 h after patient expiration. Ventilator was shut off to reduce aerosolization of viral particles at least 1 h after loss of pulse and before the sample collection. Every team member had personal protective equipment in accordance with the University policies for procedures on patients with COVID-19 (N95 mask + surgical mask, hairnet, full face shield, surgical gowns, double surgical gloves, booties). Lung biopsies were obtained after L-thoracotomy in the 5th intercostal space by our cardiothoracic surgery team. Samples were taken from the left upper lobe (LUL) and left lower lobe (LLL) and then sectioned further.

### 4.3 COVID-19 donors

Blood from COVID-19 donors was either obtained at a UC San Diego Health clinic under the approved IRB protocols of the University of California, San Diego (UCSD; 200236X) or recruited at the La Jolla Institute under IRB approved (LJI; VD-214). COVID-19 donors were California residents, who were either referred to the study by a health care provider or self-referred. Blood was collected in acid citrate dextrose (ACD) tubes (UCSD) or in EDTA tubes (LJI) and stored at room temperature prior to processing for plasma collection. Seropositivity against SARS-CoV-2 was confirmed by ELISA. At the time of enrollment, all COVID-19 donors provided written informed consent to participate in the present and future studies. Patient characteristic is listed in **Supplementary Table 5**.

### 4.4 Plasma isolation

Whole blood was collected in heparin coated blood bags (healthy unexposed donors) or in ACD tubes (COVID-19 donors) and centrifuged for 25 min at 1850 rpm to separate the cellular fraction and plasma. The plasma was then carefully removed from the cell pellet and stored at −80 °C.

### 4.5 Animal study

Lung samples from 8-week-old Syrian hamsters were generated from experiments conducted exactly as in a previously published study [14]. Animal studies were approved and performed in accordance with Scripps Research IACUC Protocol #20–0003 (PI: Tom Rogers, PMID: 32,540,903). We chose three different groups of samples: uninfected control, SARS-CoV-2 challenge after Den3 (antibody to dengue virus), and SARS-CoV-2 challenge after Anti-CoV2 (CC12.2; a potent SARS-CoV-2 neutralizing antibodies) [14].

### 4.6 Plasma IL15 cytokine ELISA

Plasma obtained from COVID-19 patients were used to quantify IL15 cytokine using ELISA MAX Deluxe set (Biolegend Cat. No. 435,104) according to the manufacturer's recommended protocol. The concentrations of IL15 cytokine were compared using Welch's *t*-test. A p < 0.05 denoted statistical significance.

### 4.7 Multiplex measurement of human serum cytokines

Human serum cytokines measurement was performed using customized Meso Scale Discovery (MSD)V-PLEX sandwich immunoassays (Cat# K151A9H-1). Human serum samples separated from peripheral blood of COVID-19 patients and healthy volunteers were analyzed using customized standard multiplex plates as per the manufacturer's instructions.

### 4.8 Immunohistochemistry

COVID-19 samples were inactivated by storing in 10% formalin for 2 days and then transferred to zinc-formalin solution for another 3 days. The deactivated tissues were transferred to 70% ethanol and cassettes were prepared for tissue sectioning. The slides containing hamster and human lung tissue sections were deparaffinized in xylene (Sigma-Aldrich Inc., MO, USA; catalog# 534,056) and rehydrated in graded alcohols to water. For IL15RA antigen retrieval, slides were immersed in Tris-EDTA buffer (pH 9.0) and boiled for 10 min at 100 °C. Slides were immersed in Tris-EDTA-Tween 20 buffer (pH 9.0) and pressure cooked for 3 min, for IL15 antigen retrieval. Endogenous peroxidase activity was blocked by incubation with 3% H2O2 for 10 min. To block non-specific protein binding 2.5% goat serum (Vector Laboratories, Burlingame, USA; catalog# S-1012) was added. Tissues were then incubated with rabbit IL15RA polyclonal antibody (1:200 dilution; proteintech®, Rosemont, IL, USA; catalog# 16,744–1-AP) for 1.5 h and mouse IL15 monoclonal antibody (1:10 dilution; Santa Cruz Biotechnology, Inc., Dallas, TX, USA; catalog# sc-8437) at room temperature in a humidified chamber and then rinsed with TBS or PBS 3x, 5 min each. Sections were incubated with goat anti-rabbit (Vector Laboratories, Burlingame, USA; catalog# MP-7401) and goat anti-mouse (Vector Laboratories, Burlingame, USA; catalog# MP-7402) secondary antibodies for 30 min at room temperature and then washed with TBS or PBS 3x, 5 min each; incubated with DAB (Vector Laboratories, Burlingame, USA; catalog# SK-4105), counterstained with hematoxylin (Sigma-Aldrich Inc., MO, USA; catalog# MHS1), dehydrated in graded alcohols, cleared in xylene, and cover slipped. Epithelial and stromal components of the lung tissue were identified by staining duplicate slides in parallel with hematoxylin and eosin (Sigma-Aldrich Inc., MO, USA; catalog# E4009) and visualizing by Leica DM1000 LED (Leica Microsystems, Germany).

### 4.9 IHC quantification

IHC images were randomly sampled at different 300 × 300 pixel regions of interest (ROI). The ROIs were analyzed using IHC Profiler [15]. IHC Profiler uses a spectral deconvolution method of DAB/hematoxylin color spectra by using optimized optical density vectors of the color deconvolution plugin for proper separation of the DAB color spectra. The histogram of the DAB intensity was divided into 4 zones: high positive (0–60), positive (61–120), low positive (121–180) and negative (181–235). High positive, positive, and low positive percentages were combined to compute the final percentage positive for each ROI. The range of values for the percent positive is compared among different experimental groups. IL15 staining showed too many ROIs with low final percent positive score. We subtracted these background noise by focusing on only ROIs with greater than 20% positive percentages.

### 4.10 RNA sequencing

RNA sequencing libraries were generated using the Illumina TruSeq Stranded Total RNA Library Prep Gold with TruSeq Unique Dual Indexes (Illumina, San Diego, CA). Samples were processed following manufacturer's instructions, except modifying RNA shear time to five minutes. Resulting libraries were multiplexed and sequenced with 100 basepair (bp) Paired End (PE100) to a depth of approximately 25–40 million reads per sample on an Illumina NovaSeq 6000 by the Institute of Genomic Medicine (IGM) at the University of California San Diego. Samples were demuxltiplexed using bcl2fastq v2.20 Conversion Software (Illumina, San Diego, CA). RNASeq data was processed using kallisto (version 0.45.0), Mesocricetus auratus genome (MesAur1.0) and human genome GRCh38 Ensembl version 94 annotation (Homo_sapiens GRCh38.94 chr_patch_hapl_scaff.gtf). Gene-level TPM values and gene annotations were computed using tximport and biomaRt R package. A custom python script was used to organize the data and log reduced using log2(TPM) if TPM > 1 and TPM - 1 if TPM <= 1. For the hamster study kallisto index was prepared on Mesocricetus_auratus.MesAur1.0.ncrna.fa.gz + Mesocricetus_auratus MesAur1.0 cdna.all.fa.gz. The raw data and processed data are deposited in Gene Expression Omnibus under accession no GSE157058 (Hamster) and GSE157059 (Ileum).

### 4.11 StepMiner analysis

StepMiner is a computational tool that identifies step-wise transitions in a time-series data [16]. StepMiner performs an adaptive regression scheme to identify the best possible step up or down based on sum-of-square errors. The steps are placed between time points at the sharpest change between low expression and high expression levels, which gives insight into the timing of the gene expression-switching event. To fit a step function, the algorithm evaluates all possible step positions, and for each position, it computes the average of the values on both sides of the step for the constant segments. An adaptive regression scheme is used that chooses the step positions that minimize the square error with the fitted data. Finally, a regression test statistic is computed as follows:

$$F\;stat=\;\frac{\sum_{i=1}^{n}{\left(\hat{X_{i}}\;-\;\bar{X}\right)}^{2}/\left(m-1\right)}{\sum_{i=1}^{n}{\left(X_{i}-\;\hat{X_{i}}\right)}^{2}/\left(n-m\right)}$$

Where $X_{i}$ fori=1 ton are the values, $\hat{X_{i}}$ fori=1 ton are fitted values. m is the degrees of freedom used for the adaptive regression analysis. $\bar{X}$ is the average of all the values: $\bar{X}=\;\frac{1}{n}*\;\sum_{j=1}^{n}X_{j}.\;$For a step position at k, the fitted values $\hat{X_{l}}$ are computed by using $\frac{1}{k}*\;\sum_{j=1}^{n}X_{j}$ fori=1 tok and $\frac{1}{\left(n-k\right)}*\;\sum_{j=k+1}^{n}X_{j}$ fori=k+1 ton.

### 4.12 Boolean analysis

**Fig. 1:**
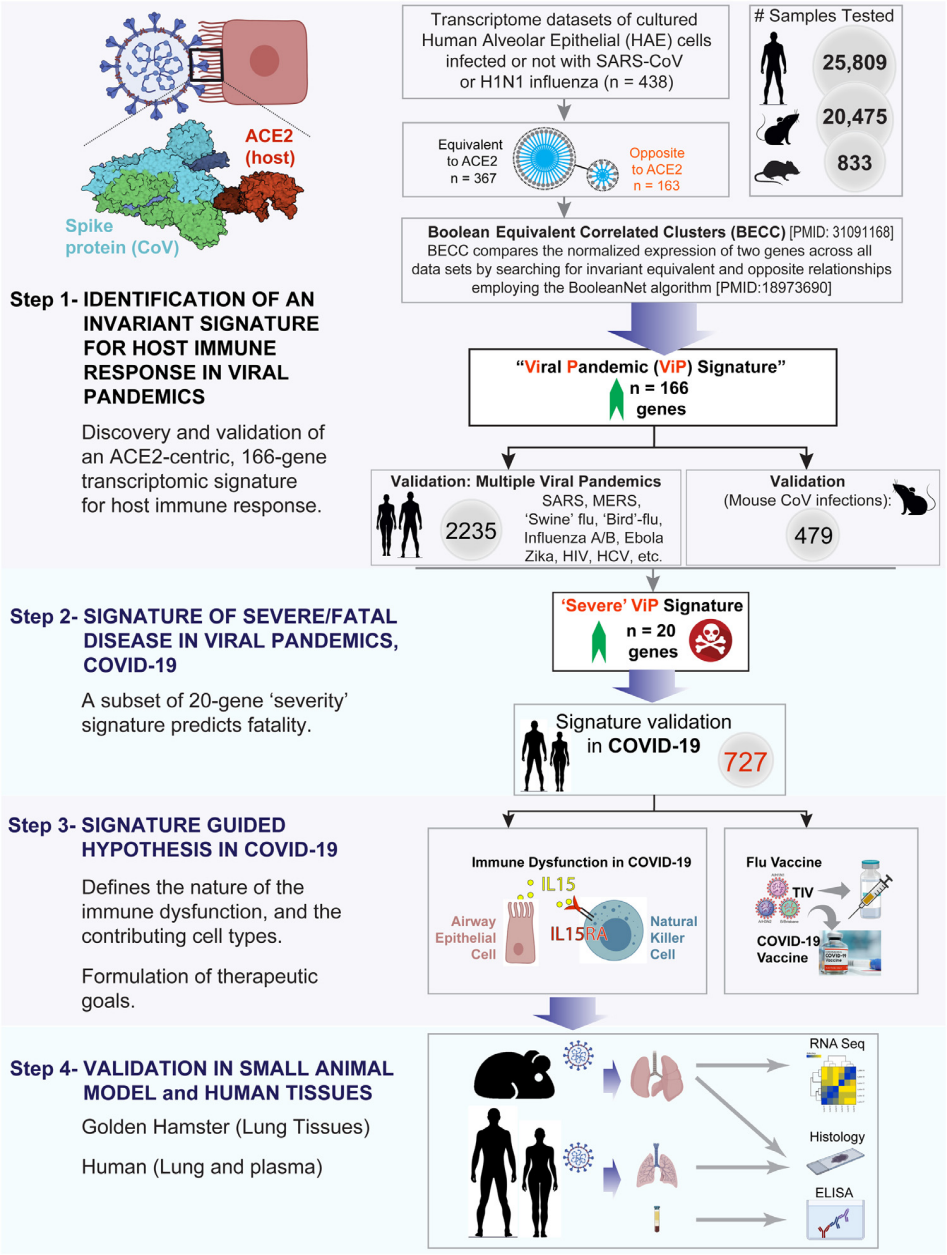
Study design. (*From top to bottom*) *Step 1*: A database containing > 45,000 human, mouse and rat gene–expression data was mined to identify and validate an invariant signature for host response to viral pandemic (*ViP*) infection. ACE2, the portal for SARS-CoV-2 entry/uptake, was used as a ‘seed’ gene and Boolean Equivalent Correlated Clusters (BECC) was used as the computational method to identify gene clusters that share invariant relationships with ACE2. Once defined, these gene clusters (a.k.a., ‘*ViP signature*’) were subsequently validated across multiple human and murine models of pandemic viral infection. *Step 2:* A subset of 20 genes from the *ViP* signature was selected that was strongly associated with severity of viral infection. These genes were validated in other cohorts to establish the ‘Severe’ *ViP* signature. Both 166- and 20-gene *ViP* signatures were validated on COVID-19 datasets. *Step 3*: Cross-validation studies in numerous other datasets helped- (i) define the nature (ii) and source of the cytokine storm in COVID-19, (iii) gain insights into the immunopathology of fatal disease, and (iv) set precise therapeutic goals. *Step 4*: Findings in step 3 were validated in hamsters and in a cohort of COVID-19 patients. A comprehensive catalog of the datasets analyzed in this work can be found in **Supplementary Table 1**.

**Fig. 2:**
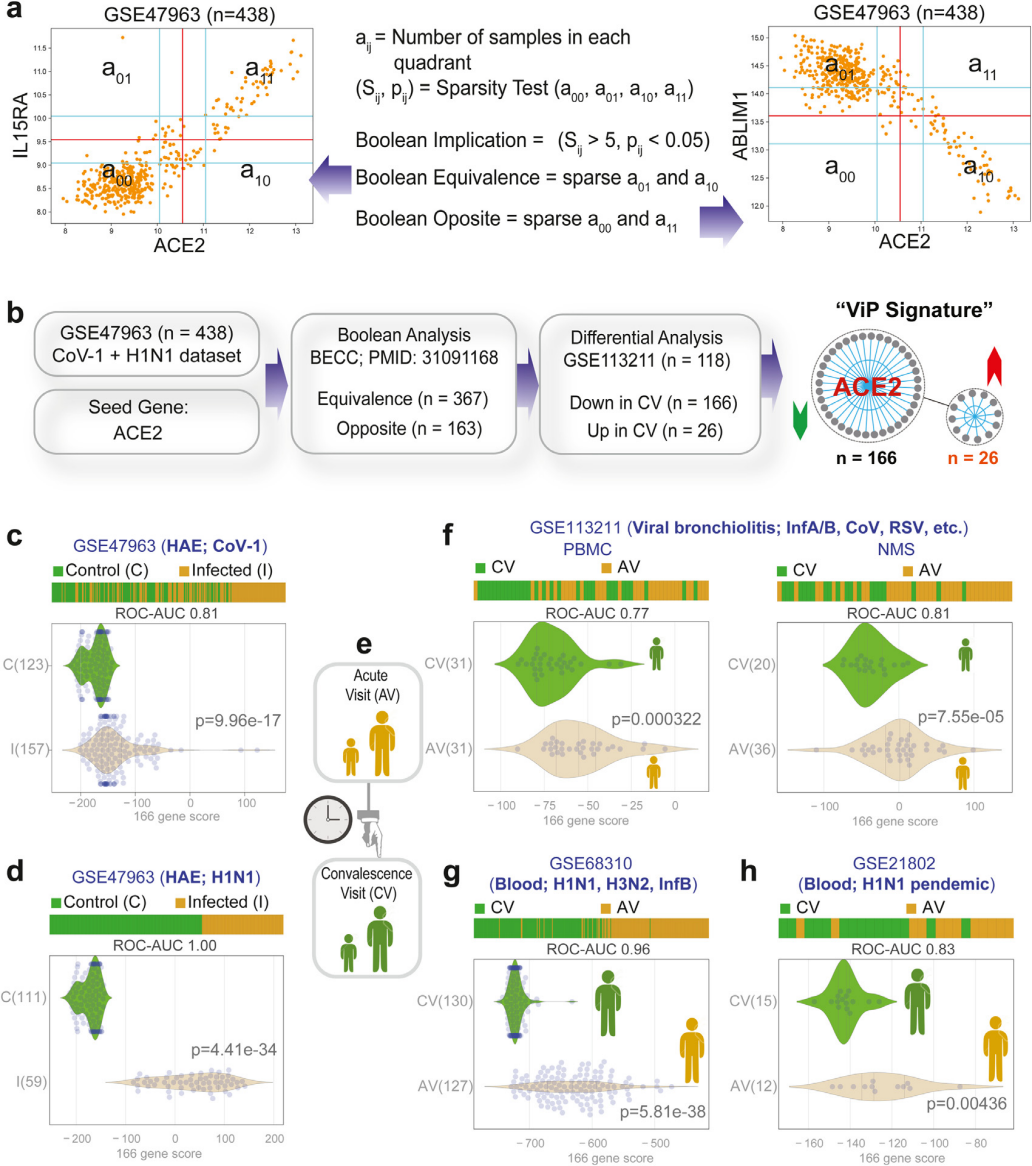
Identification and validation of an invariant ACE2-centric signature of host response to viral infections. **(a)** Computational approach to identify Boolean Equivalent and Opposite relationships. Number of samples in all four quadrants are used to compute two parameters (S, p). *S* > 5 and *p* < 0.05 is used to identify sparse quadrant. Equivalent relationships are discovered when top-left and bottom-right quadrants are sparse (*left*). Opposite relationships are discovered when top-right and bottom-left quadrants are sparse (*right*). **(b)** Schematic displaying the key computational steps and findings leading to the identification of the 166-gene host response signature using ACE2 as a ‘seed’ gene. See also **Table S2. (c)** Bar and violin plots displaying sample rank order (i.e., classification) of SARS-CoV-1-infected samples and distribution of the 166 gene-based signature in the test dataset (GSE47963, *in vitro* infections of human airway epithelial cells). ROC-AUC values of infected samples classifications are shown below each bar plot unless otherwise stated. **(d)** Analysis of H1N1-infected samples compared to uninfected controls using the 166-gene signature like C. **(e)** Classification of patient samples used in datasets **f-h** based on their time of collection either during ‘Acute Visit’ (AV) in the setting of an acute respiratory viral infection and ‘Convalescence Visit’ (CV) after recovery. **(f)** Analysis of PBMC samples from children (GSE113211, left) and nasal mucosal scrapings (NMS, GSE113211, right). **(g)** Analysis of peripheral blood from adults (GSE68310). **(h)** Analysis of patient samples collected during the swine flu pandemic (GSE21802).

*Boolean logic* is a simple mathematic relationship of two values, i.e., high/low, 1/0, or positive/negative. The Boolean analysis of gene expression data requires the conversion of expression levels into two possible values. The *StepMiner* algorithm is reused to perform Boolean analysis of gene expression data [17]. *The Boolean analysis* is a statistical approach which creates binary logical inferences that explain the relationships between phenomena. Boolean analysis is performed to determine the relationship between the expression levels of pairs of genes. The *StepMiner* algorithm is applied to gene expression levels to convert them into Boolean values (high and low). In this algorithm, first the expression values are sorted from low to high and a rising step function is fitted to the series to identify the threshold. Middle of the step is used as the StepMiner threshold. This threshold is used to convert gene expression values into Boolean values. A noise margin of 2-fold change is applied around the threshold to determine intermediate values, and these values are ignored during Boolean analysis. In a scatter plot, there are four possible quadrants based on Boolean values: (low, low), (low, high), (high, low), (high, high). A Boolean implication relationship is observed if any one of the four possible quadrants or two diagonally opposite quadrants are sparsely populated. Based on this rule, there are six kinds of Boolean implication relationships. Two of them are symmetric: equivalent (corresponding to the positively correlated genes), opposite (corresponding to the highly negatively correlated genes). Four of the Boolean relationships are asymmetric and each corresponds to one sparse quadrant: (low => low), (high => low), (low => high), (high => high). BooleanNet statistics (Fig. 2**a**) is used to assess the sparsity of a quadrant and the significance of the Boolean implication relationships [17,18]. Given a pair of genes A and B, four quadrants are identified by using the StepMiner thresholds on A and B by ignoring the Intermediate values defined by the noise margin of 2-fold change (+/- 0.5 around StepMiner threshold). Number of samples in each quadrant are defined as a_00_, a_01_, a_10_, and a_11_ (Fig. 1A) which is different from X in the previous equation of F stat. Total number of samples where gene expression values for A and B are low is computed using the following equations.

$$nA_{low}=\;\left(a_{00}+\;a_{01}\right),\;nB_{low}=\;\left(a_{00}+\;a_{10}\right)$$

Total number of samples considered is computed using following equation.

$$total=\;a_{00}+\;a_{01}+\;a_{10}+\;a_{11}$$

Expected number of samples in each quadrant is computed by assuming independence between A and B. For example, expected number of samples in the bottom left quadrant e_00_ = $\hat{n}$ is computed as probability of A low ((a_00_ + a_01_)/total) multiplied by probability of B low ((a_00_ + a_10_)/total) multiplied by total number of samples. Following equation is used to compute the expected number of samples.

$$n=\;a_{ij},\;\hat{n}=\;\left(nA_{low}/total*\;nB_{low}/total\right)*total$$

To check whether a quadrant is sparse, a statistical test for (e_00_ > a_00_) or ($\hat{n}>n)$ is performed by computing S_00_ and p_00_ using following equations. A quadrant is considered sparse if S_00_ is high ($\hat{n}>n)$ and p_00_ is small.

$$S_{ij}=\;\frac{\hat{n}-n}{\sqrt{\hat{n}}}$$

$$p_{00}=\;\frac{1}{2}\;\left(\frac{a_{00}}{\left(a_{00}+\;a_{01}\right)}+\;\frac{a_{00}}{\left(a_{00}+a_{10}\right)}\right)$$

A suitable threshold is chosen for S_00_ > sThr and p_00_ < pThr to check sparse quadrant. A Boolean implication relationship is identified when a sparse quadrant is discovered using following equation.

***Boolean Implication*** = (*S_ij_* > sThr, *p_ij_* < pThr)

A relationship is called Boolean equivalent if top-left and bottom-right quadrants are sparse.

$$Equivalent=\;\left(S_{01}>\;sThr,\;P_{01}\langle \;pThr,\;S_{10}\rangle \;sThr,\;P_{10}<\;pThr\right)$$

Boolean opposite relationships have sparse top-right (a_11_) and bottom-left (a_00_) quadrants.

$$\;Opposite=\;\left(S_{00}>\;sThr,\;P_{00}\langle \;pThr,\;S_{11}\rangle \;sThr,\;P_{11}<\;pThr\right)$$

Boolean equivalent and opposite are symmetric relationship because the relationship from A to B is same as from B to A. Asymmetric relationship forms when there is only one quadrant sparse (A low => *B* low: top-left; A low => *B* high: bottom-left; A high=> *B* high: bottom-right; A high => *B* low: top-right). These relationships are asymmetric because the relationship from A to B is different from B to A. For example, A low => *B* low and B low => *A* low are two different relationships.

A low => *B* high is discovered if the bottom-left (a_00_) quadrant is sparse and this relationship satisfies following conditions.

*A low => B high* = ($S_{00}>\;sThr,\;P_{00}<\;pThr$)

Similarly, A low => *B* low is identified if the top-left (a_01_) quadrant is sparse.

*A low => B low* = ($S_{01}>\;sThr,\;P_{01}<\;pThr$)

A high => *B* high Boolean implication is established if the bottom-right (a_10_) quadrant is sparse as described below.

*A high => B high* = ($S_{10}>\;sThr,\;P_{10}<\;pThr$)

Boolean implication A high => *B* low is found if the top-right (a_11_) quadrant is sparse using following equation.

*A high => B low* = ($S_{11}>\;sThr,\;P_{11}<\;pThr$)

For each quadrant a statistic S_ij_ and an error rate p_ij_ is computed. S_ij_ > sThr and p_ij_ < pThr are the thresholds used on the BooleanNet statistics to identify Boolean implication relationships. False discovery rate is computed by randomly shuffling each gene and computing the ratio of the number of Boolean implication relationship discovered in the randomized dataset and original dataset.

Boolean analyses in the test dataset GSE47963 uses a threshold of sThr = 5 and pThr = 0.05. Boolean analysis on the large normal lung dataset GSE23546 uses a threshold of sThr = 6 and pThr = 0.1. These thresholds are more stringent compared to previously used thresholds sThr = 3 and pThr = 0.1 for BooleanNet [17,19,20] to focus on the strong candidates. These thresholds are so stringent that the false discovery rate was 0 which means no Boolean implication relationships were discovered in the randomly permuted data.

### 4.13 BECC (Boolean equivalent correlated clusters) analysis

BECC analysis [20] is based on Boolean Equivalent relationships, pair-wise correlation and linear regression analysis. BECC analysis begins with a seed gene. We used ACE2 as a seed gene in this paper. BECC analysis identified a set of genes Boolean Equivalent to ACE2 and Boolean Opposite to ACE2 using the BooleanNet statistic described above.

The BECC algorithm identified 367 genes ‘Boolean Equivalent’ and 163 genes ‘Boolean Opposite’ to the ACE2 gene. Reactome pathway analyses on both clusters showed that the 367-gene ACE2-equivalent cluster was enriched in viral response pathways and processes, whereas the 163-gene ACE2-opposite cluster represented housekeeping processes, implying that ACE2 and its related genes are drivers of host response in the setting of viral infections. These clusters were subsequently filtered using differential analysis on another dataset [GSE113211 (*n* = 118); Fig. 2**b**] that profiled heterogeneous immunophenotypes of children with viral bronchiolitis (confirmed positive for the virus in ~100% patients; of which 25% were infected with Influenza/Para-Influenza and 14.8% with human CoV). We chose GSE113211 (*n* = 118) dataset to filter *ViP* genes because this is the only high-quality large *in vivo* dataset available with clinical annotation on two different tissue types: nasal mucosal scrapings (NMS) and PBMC. Transcriptomes were analyzed in nasal mucosal scrapings (NMS) and PBMC samples taken during an acute visit (AV) and during a subsequent visit at convalescence (CV) [21]. Of the 367 ACE2-equivalent genes, 166 genes (**Table S2**; 1–1) retained the “Boolean Equivalent” relationship with ACE2 *and* their expression was downregulated during the convalescence visit. Of the 163 ACE2-opposite genes, 26 genes (**Table S2;** 2–1) retained “Boolean Opposite” relationships with ACE2 *and* their expression were upregulated during the convalescence visit. All subsequent analyses were performed using the 166 –gene signature that had Boolean Equivalent relationship with ACE2 and that was down-regulated during a convalescent visit after acute viral bronchiolitis.

A gene signature score is computed using the 166-genes that were equivalent to ACE2 which is used to order the sample. To compute the *ViP* signature, first the genes present in this list were normalized according to a modified Z-score approach centered around StepMiner threshold (formula = (expr -SThr)/3*stddev). The normalized expression values for every probeset for 166 genes were added together to create the final *ViP* signature. The samples were ordered based on the final *ViP* signature. To compute the *severe-ViP* signature, 166 genes were first ordered using T test between the mild vs severe cases in GSE101702 dataset, and top 20 genes (**Table S2;** 3–1) were selected from this list. We chose GSE101702 dataset to select *Severe ViP* genes because this is the only high-quality large dataset available with clinical severity annotation.

To test the significance of the *ViP* and *Severe ViP* genes, we subsampled GSE47963 dataset to see if similar number of genes appear after BECC analysis. We selected 250 samples from 438 total number of samples 10,000 times randomly and performed BECC on them. Boolean analysis on the original GSE47963 used thresholds of sThr = 5 and pThr = 0.05. These thresholds need to be adjusted when the number of samples are reduced. For the BECC analysis on the 250 randomly selected samples we used thresholds of sThr = 4 and pThr = 0.06 which discovers around 367 genes on average. Analysis of the genes discovered in the 250 subsampled datasets revealed that on average 87% (321 out 367) emerge again and 41 new genes appear. In this test, on average 90% (150 out of 166) *ViP* genes and 90% (18 out of 20) *Sever ViP* genes emerge again. 17 new *ViP* genes and 2 new *Severe ViP* genes appear. These results suggests that there are about 10% variation in the genes which is a reasonable criterion for robustness.

To understand how the downstream results will change if a different seed gene was used, we carry out following analyses. If we choose seed genes from the 166 genes, more than 75% of the genes matched more than 75% with the 166-gene signature. When using an immune related gene as ‘seed’, e.g., LMO2 matches 92% (130/141) with the ACE2-centric 166 genes. Similarly, other relevant immune genes such as TRIM26, IL15RA, HLA-E, HLA-H, HLA-B, TLR2, and TLR3 as seed genes individually matched more than 82% with the ACE2-centric 166 genes.

### 4.14 Single cell RNASeq data analysis

Single Cell RNASeq data from GSE145926 and GSE150728 was downloaded from Gene Expression Omnibus (GEO) in the HDF5 Feature Barcode Matrix Format. The filtered barcode data matrix was processed using Seurat v3 R package [22]. B cells (CD19, MS4A1, CD79A), T cells (CD3D, CD3E, CD3G), CD4 T cells (CCR7, CD4, IL7R, FOXP3, IL2RA), CD8 T cells (CD8A, CD8B), Natural killer cells (KLRF1), Macs Monos DCs (TYROBP, FCER1G), Epithelial (SFTPA1, SFTPB, AGER, AQP4, SFTPC, SCGB3A2, KRT5, CYP2F1, CCDC153, TPPP3) cells were identified using relevant gene markers using SCINA algorithm [23]. Pseudo bulk datasets were prepared by adding counts from the different cell subtypes and normalized using log(CPM+1).

### 4.15 AI guided discovery of invariant host response

BECC requires the depth of Boolean equivalent relationship as a parameter. For example, if ACE2 is Equivalent to X and X is Equivalent to Y but ACE2 is not necessarily equivalent to Y, depth of X is 1 and Y is 2. The depth controls how much the list of genes that are Boolean equivalent to ACE2 is expanded. This list of genes is converted to a gene expression score based on average of the normalized gene expression values as mentioned before. The strength of classification of uninfected and infected samples using this score is computed by the ROC-AUC measurement. We performed a regression to identify the best depth that predicts uninfected vs infected samples in the cohort GSE47963 (*n* = 438). We tested how the gene expression score distinguish uninfected and infected samples as they are annotated in many other independent datasets. Our confidence on the host response being invariant depend on having this test pass in all properly annotated cohorts without exceptions.

### 4.16 Survival outcome in COVID-19

Hospital-free days analysis (45 days followup) of COVID-19 patients (GSE157103) limited to less than 70 years old using *sViP* signature (low and high group) is analyzed using Kaplan-Meier and Cox-proportional hazard approach. The threshold to separate high and low group was computed using StepMiner determined threshold + *a* noise margin. The noise margin for *sViP* signature was computed by computing the total dynamic range (max – min) divided by 65 to bring it to comparable levels of two-fold change noise margin seen in gene expression datasets. For the IL15 transcript analysis samples were limited to only males with less than 70 years old. IL15 transcripts were divided into high, intermediate and low levels by using StepMiner threshold +/- noise margin 1 which is two-fold change in log scale. Low levels of IL15 were associated with unusually adverse outcome. High and intermediate levels were compared to demonstrate the significance of IL15 in the context of our manuscript.

### 4.17 Statistical analyses

Boolean analysis and other statistical approaches are covered in detail above. Briefly, the StepMiner algorithm [16], BooleanNet statistics [17], and BECC (Boolean Equivalent Correlated Clusters) [20] are used to perform Boolean analyses. Gene signature is used to classify sample categories and the performance of the multi-class classification is measured by ROC-AUC (Receiver Operating Characteristics Area Under The Curve) values. A color-coded bar plot is combined with a density plot to visualize the gene signature-based classification. Bubble plots of ROC-AUC values (radius of circles are based on the ROC-AUC) demonstrating the direction of gene regulation (Up, red; Down, blue) for the classification based on the 20 gene *severe ViP* signature and 166 gene *ViP* signature is visualized side by side. All statistical tests were performed using R version 3.2.3 (2015–12–10). Standard t-tests were performed using python scipy.stats.ttest_ind package (version 0.19.0) with Welch's Two Sample *t*-test (unpaired, unequal variance (equal_var=False), and unequal sample size) parameters. Multiple hypothesis correction were performed by adjusting *p* values with statsmodels.stats.multitest.multipletests (fdr_bh: Benjamini/Hochberg principles). The results were independently validated with R statistical software (R version 3.6.1; 2019–07–05). Pathway analysis of gene lists were carried out via the Reactome database and algorithm [24]. Reactome identifies signaling and metabolic molecules and organizes their relations into biological pathways and processes. Kaplan-Meier analysis is performed using lifelines python package version 0.14.6. Violin, Swarm and Bubble plots are created using python seaborn package version 0.10.1.

*Sample size estimation:* Effect size (the magnitude of the difference between groups divided by the standard deviation) for IL15 measurement is estimated as 1/0.7 from GSE157103. In order to have an 80% power (1-β = 0.8) to detect a statistically significant difference (α = 0.05) between high and low groups of patients, we need around 8 patients in each group.

*Ethics statement:* Animal studies were approved and performed in accordance with Scripps Research IACUC Protocol #20–0003 (PI: Tom Rogers, PMID: 32,540,903) [14]. Blood from COVID-19 donors was either obtained at a UC San Diego Health clinic under the approved IRB protocols of the University of California, San Diego (UCSD; 200236X) or recruited at the La Jolla Institute under IRB approved (LJI; VD-214). COVID-19 donors were California residents who were either referred to the study by a health care provider or self-referred. The lung specimens from the COVID 19 positive human subjects were collected using autopsy (study was IRB Exempt). All donations to this trial were obtained after telephone consent followed by written email confirmation with next of kin/power of attorney per California state law (no in-person visitation could be allowed into our COVID-19 ICU during the pandemic). The team member followed the CDC guidelines for COVID19 and the autopsy procedures [25,26].

*Role of funding source:* Funders of the study had no role in study design, data collection, data analyses, interpretation, or writing of report.

## 5 Results and discussion

### 5.1 An ACE2-centric study design

To identify and validate an invariant (universal) gene signature of host response in COVID-19, we mined more than 45,000 publicly available datasets of viral pandemics across three species (human, mouse and rats) (***Step 1***; Fig. 1). Three relatively widely accepted facts shaped our approach using Angiotensin-converting enzyme 2 (ACE2) as ‘seed’ gene in our computational studies: *(i)* ACE2 is the most well-known portal for SARS-CoV-2 entry into the host cell [27,28]; its expression in cell lines correlates with the expression of innate immune genes [29] and susceptibility to SARS-CoV spike protein-driven entry [30,31], and its depletion in mice abrogates SARS-CoV infection [32]; *(ii)* ACE2 is a potent negative regulator of the renin–angiotensin aldosterone system (RAAS) [33]; without such restraint, the RAAS contributes to exuberant inflammation in the setting of infections [34]; and finally, *(iii)* although the mechanism through which ACE2 suppresses inflammatory response remains poorly understood, accumulating evidence indicates that infections perturb ACE2 activity, allowing for uncontrolled inflammation [35], [36], [37], [38], [39], [40], [41], [42], [43].

As ***Step 2*** (Fig. 1), we validated the signature in several human and mouse datasets of viral pandemics, and a subset of genes was identified and validated as indicators of disease severity. The signatures were then validated in SARS-CoV-2-infected cells and tissues and to explore the nature, extent and cell of origin of host response in mild and fatal COVID-19.

As ***Step 3*** (Fig. 1), the gene signatures were prospectively used to navigate the uncharted territory of COVID-19 and pinpoint immunopathologic mechanisms, which revealed the nature (IL15), source (airway epithelium), intensity (quantitative measure) and consequence (NK cell senescence) of the cytokine storm and helped objectively formulate precise therapeutic goals to reduce the severity of COVID-19.

As ***Step 4*** (Fig. 1), the gene signature and the mechanism of action (IL15/IL15RA) were validated in lung tissues from SARS-CoV-2 challenged golden hamster using RNASeq and IHC. In addition, precise therapeutic goal was validated in the SARS-CoV-2-challenged golden hamster model. The mechanism of action (IL15/IL15RA) was also validated by ELISA in plasma and IHC in lung tissues from UCSD COVID-19 cohort participants.

### 5.2 A shared host response signature in respiratory viral pandemics

Because publicly available transcriptomic datasets from SARS-CoV-2-infected samples are still relatively few, any conclusion drawn from so few samples using any computational methodology is likely to lack robustness. We chose to use an informatics approach, i.e., Boolean Equivalent Correlated Clusters (BECC) [20], which can identify fundamental invariant (universal) gene expression relationships underlying any biological domain; in this case, we selected the biological domain of '*respiratory viral pandemics*’. BECC enables comparison of the normalized expression of two genes across all datasets by searching for two sparsely populated, diagonally opposite quadrants out of four possible quadrants (high-low and low-high), employing the BooleanNet algorithm [17]. There are six potential gene relationships assessed by BooleanNet: two symmetric (Equivalent and Opposite; Fig. 2**a**) and four asymmetric [17]. Two genes are considered “Boolean Equivalent” if they are positively correlated with only high-high and low-low gene expression values. Two genes are considered “Boolean Opposite” if they are negatively correlated with only high-low and low-high gene expression values. Asymmetric Boolean implications result when there is only one sparsely populated quadrant. The BECC algorithm focuses exclusively on “Boolean Equivalent” relationships to identify potentially functionally related gene sets. Once identified, these invariant relationships have been shown to spur new fundamental discoveries [44,45], with translational potential [46], and most importantly, offer insights that aid the navigation of uncharted territories where nothing may be known [47,48].

We used GSE47963 [human airway epithelial (HAE) cultures with H1N1 and SARS-CoV infections; *n* = 438] as a ‘test’ dataset, which was comprised of human airway epithelial cell samples (HAE) infected *in vitro* with the causative agents of the 2009 ‘swine flu’ (influenza A-H1N1; a triple recombination of human, avian, and swine influenza viruses [49], [50], [51]) and the 2002 Severe acute respiratory syndrome (SARS-CoV-1) [52] outbreaks (Fig. 2**b**). These datasets were chosen now, and other datasets were prioritized later in the study, e.g., H5N1 (the causative agent of the avian flu in 2006–06 [53] and MERS-CoV (the causative agent of Middle East respiratory syndrome in 2012 [54]) based upon the fact that they *all* contributed to outbreaks that are characterized by acute respiratory syndromes with high case-fatality rates [27].

ACE2 is used as a ‘seed’ to identify other genes that have ‘Boolean Equivalent’ and ‘Boolean Opposite’ relationships with ACE2. These genes were subsequently filtered using differential analysis on another dataset [GSE113211 (*n* = 118); Fig. 2**b**] that profiled heterogeneous immunophenotypes of children with viral bronchiolitis (confirmed positive for the virus in ~100% patients; of which 25% were infected with Influenza/Para-Influenza and 14.8% with human CoV). Transcriptomes were analyzed in nasal mucosal scrapings (NMS) and PBMC samples taken during an acute visit (AV) and during a subsequent visit at convalescence (CV) [21]. 166 genes (**Table S2**; 1–1) retained the “Boolean Equivalent” relationship with ACE2 *and* their expression was downregulated during the convalescence visit. 26 genes (**Table S2;** 2–1) retained “Boolean Opposite” relationships with ACE2 *and* their expression was upregulated during the convalescence visit. All subsequent analyses were performed using the 166 –gene signature that had Boolean Equivalent relationship with ACE2 and that was down-regulated during a convalescent visit after acute viral bronchiolitis.

First, the 166-gene signature was evaluated in the test dataset– it was used to rank order the samples and test for phenotype classification using a receiver operating characteristic curve [ROC curve; the area under this curve (AUC) represents degree or measure of separability] and displayed such classification using violin plots (Fig. 2**c,d**). The signature classified the uninfected vs. infected samples with reasonable accuracy in the setting of SARS-CoV-1 infection (ROC-AUC = 0.81, Fig. 2**c**). It also classified perfectly in the setting of H1N1 infection (ROC-AUC = 1.00, Fig. 2**d**). Good classification was observed between samples from the acute visit (AV) and convalescence visit (CV) in children (test dataset; GSE113211; Fig. 2**e,f**, *left*), as well as two independent adult cohorts (validation datasets that were generated in two prospective studies [55,56]; Fig. 2**g,h**). All the patients in these cohorts were infected with respiratory viruses; in one cohort, ~45% were documented infections with pandemic Influenza strains H1N1 and H3N2 (GSE68310), whereas 100% of the patients in the other were victims of the H1N1 pandemic of 2009 (GSE21802). Regardless of the heterogeneity of these validation cohorts, the classification score using the 166-gene signature remained strong in both datasets (ROC-AUC = 0.83 - 0.96). Findings indicate that the viral pandemic signature was conserved among numerous respiratory viral pandemics, and for that reason, we christened it the ‘*Vi*ral *P*andemic’ (*ViP*) Signature.

### 5.3 The *ViP* signature defines the ‘Cytokine storm’ in viral pandemics

**Fig. 3:**
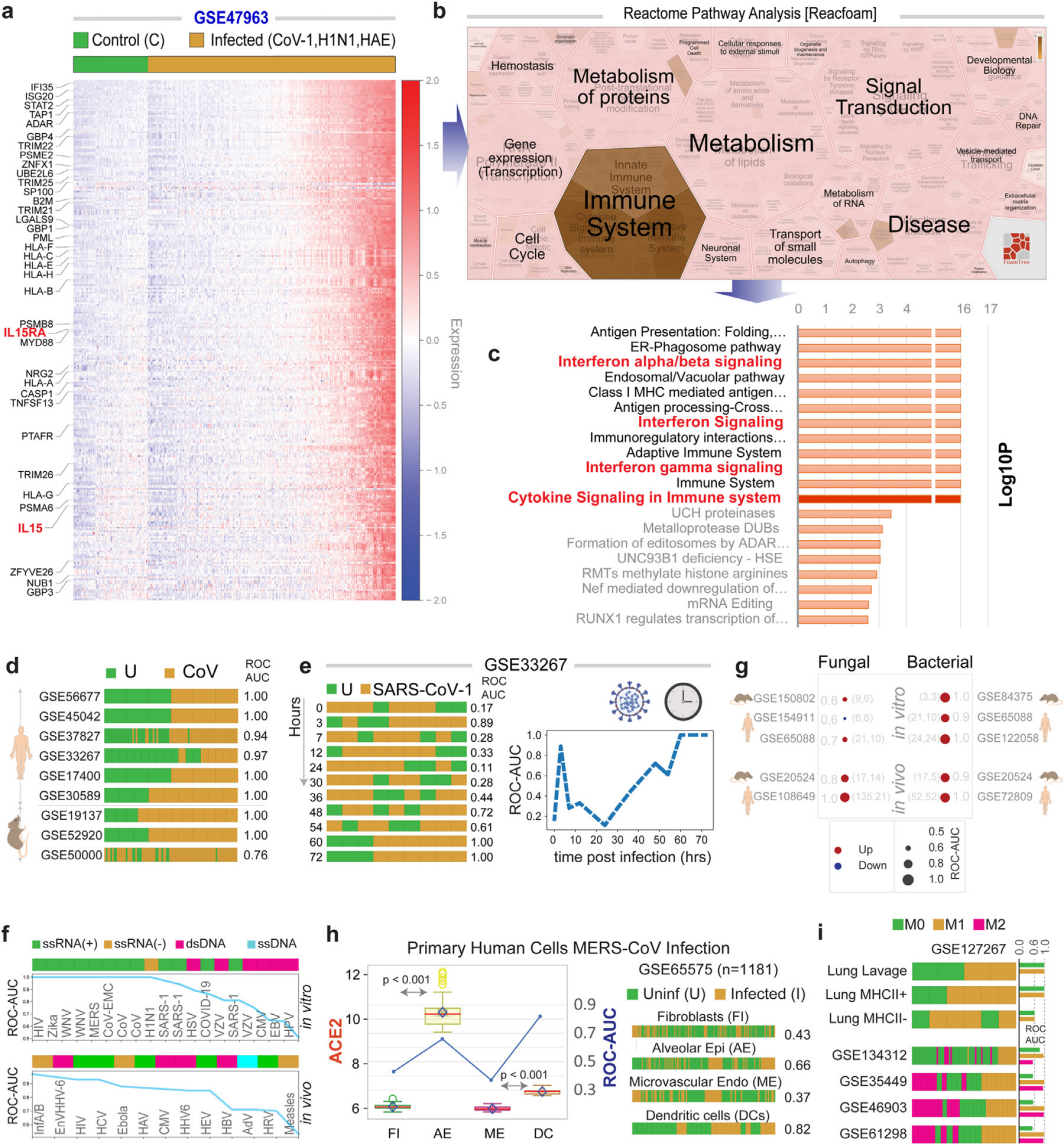
Validation of the *ViP* signatures in global pandemic viral infections. **(a)** Heatmap of the 166-gene signature on test dataset (GSE47963, *in vitro* infections of human airway epithelial cells). Genes that are involved in cytokine signaling in immune system are highlighted in the left. **(b)** ReacFoam analysis on the 166-gene signature that visualizes genome-wide pathway analysis based on Voronoi tessellation. **(c)** Reactome pathway analysis of the 166 genes in the *Vip* signature. **(d)***ViP* signature-based classification of CoV-infected samples (CoV) from uninfected controls (U) in diverse human and mouse datasets. **(e)** Time course of CoV infection shows that the *ViP* host-response signature is slowly induced in very late (48–72 h) in Calu-3 cells infected with SARS-CoV-1. **(f)** The accuracy (Y axis; ROC AUC) of the signature to classify viral infections differs between RNA viruses and DNA viruses (*X* axis) in *in vitro* system (top). However, they are indistinguishable in *in vivo* system (bottom). See also **Table S3** and **Fig S1. (g)***ViP* signature-based classification of human and murine samples with fungal or bacterial infections in either *in vitro* or *in vivo* settings. **(h)** The signature captures host response to CoV infection in human primary lung alveolar epithelial cells (AE) and dendritic cells (DC) better than in Fibroblasts (FI) and Endothelial (ME) cells. The accuracy of classification (ROC-AUC) strongly correlates with ACE2 expression in these cells. **(i)** Classification of macrophage polarization states ‘reactive’ (M1 polarized), unstimulated M0 and tolerant M2-like samples using the 166-gene *ViP* signature across diverse datasets.

Reactome analyses on the 166 genes showed that the signature was largely enriched for genes within the immune system pathways, e.g., interferon and cytokine signaling, cellular processes that are critical for an innate immune response such as the ER-phagosome pathway and antigen processing and presentation, and finally the adaptive immune system (Fig. 3**a–c**). In other words, the signature reflected a typical host immune response that is expected during any viral infection. This is not surprising because an overzealous host immune response, i.e., a ‘cytokine storm’ is shared among all respiratory viral pandemics (Influenza, avian and swine flu) [57] and severe COVID-19 patients who succumb to the disease [6]. However, there were 3 surprising factors: (i) This signature and reactome profile emerged using ACE2 as a ‘seed’ gene, which is not the receptor for influenza strains to enter into host cells. (ii) It is also noteworthy that despite filtration through two unrelated datasets (Fig. 2**b**), one *in vitro* and another *in vivo*, and the reduction in the number of genes in the ACE2-equivalent cluster during such iterative refinement, the pathways/processes represented in the 166-gene cluster (**Table S2**; 1–2) remained virtually unchanged. (iii) The only cytokine/receptor pair that emerged in this 166-gene cluster was interleukin-15 (IL15/IL15RA; Fig. 3**a, c**), indicating that transcripts of this cytokine are invariably equivalent with ACE2 expression across all datasets analyzed. Findings are in keeping with the well-established role of IL15 in both the pathogenesis [58] and the severity [59] of virus-induced lung injury. They are also consistent with the fact that IL15-/- mice are protected from lethal influenza [60].

Next, we tested this 166-gene signature in datasets of samples infected with viruses that have either caused pandemics in the past (SARS-CoV-1, MERS, Ebola, Zika, etc.) or continue to do so at present (Influenza A/B, HIV, HCV, etc.). The signature perfectly classified uninfected and infected samples (ROC-AUC = 1.00; Fig. 3**d**) in four humans (GSE56677, GSE45042, GSE17400, GSE30589) and two mouse SARS-CoV1 and MERS-CoV datasets (GSE19137, GSE52920). It also performed reasonably well in two other human and one mouse datasets (ROC-AUC ranging between 0.76–0.97; GSE37827, GSE33267, GSE50000; Fig. 3**d**). Analysis of a time course of infection with SARS-CoV-1 (GSE33267; Fig. 3**e**) revealed that classification of infected samples improved over time, beginning at 48 h and reaching perfection (ROC AUC = 1.00) at 60–72 h, which is consistent with epidemiologic findings in prior acute respiratory viral pandemics (SARS and MERS) have average incubation periods ranging ~2–7 days, which can sometimes last up to ~10–14 d. Among datasets with curated samples representing other viral outbreaks and/or pandemics that are neither respiratory nor acute, we found that classification scores for RNA viruses were significantly better compared to DNA viruses in *in vitro* systems (Fig. 3**f** top, **Fig. S1a**), especially for those that share clathrin-dependent endocytic methods to breach host cells (**Table S3**). However, the classification scores were indistinguishable between RNA and DNA viruses in *in vivo* studies (Fig. 3**f** bottom, **Fig. S1a-b**). These results indicate that the 166-gene signature is shared among all viral infections, and not specific to respiratory viral pathogens.

Notably, the 166-gene host response signature was not specific for viral infections *per se*; it also performed well in classifying samples with bacterial infections, both *in vitro* and *in vivo*, and fungal infections *in vivo* (Fig. 3**g**). These findings were not surprising because the prominent overrepresentation of interferon signaling that is captured within the signature (Fig. 3**c**) is widely accepted as a shared fundamental aspect of host defense response during any infection [61]. Despite such apparent promiscuity, what is noteworthy is that the *ViP* signatures were relatively specific for infections/inflammation (**Fig. S3**). The signature also implicated the epithelial and myeloid cells, but not ECs and fibroblasts contribute to host immune response because the classification scores were better for airway epithelial cells (AE) and dendritic cells (DC) compared to fibroblasts (FI) and microvascular endothelial cells (ME) (ROC-AUC: 0.66, 0.82 vs 0.43, 0.37; Fig. 3**h**; *left*). These scores correlated well with ACE2 expression in these different cell types (*p* < 0.001; Fig. 3**h**; *right*), raising the possibility that viral entry through the engagement of ACE2 and the induction of ACE2-equivalent host genes may be intertwined. That myeloid cells are major contributors to this signature was confirmed in five independent datasets; the 166-gene signature distinguished ‘reactive’ (M1-polarized) macrophages in them all (Fig. 3**i**).

Together, these findings indicate that the ACE2-equivalent 166-gene signature is of broader relevance than just coronaviruses; the signature captures core fundamentals of host innate immune responses seen not just in respiratory viral pandemics, but viral, bacterial and even fungal infections. The airway epithelial cells and cells of myeloid lineages (DCs and macrophages) appear to be major contributors to the *ViP* signature.

### 5.4 A 20-gene subset within the *ViP* signature detects disease severity

**Fig. 4:**
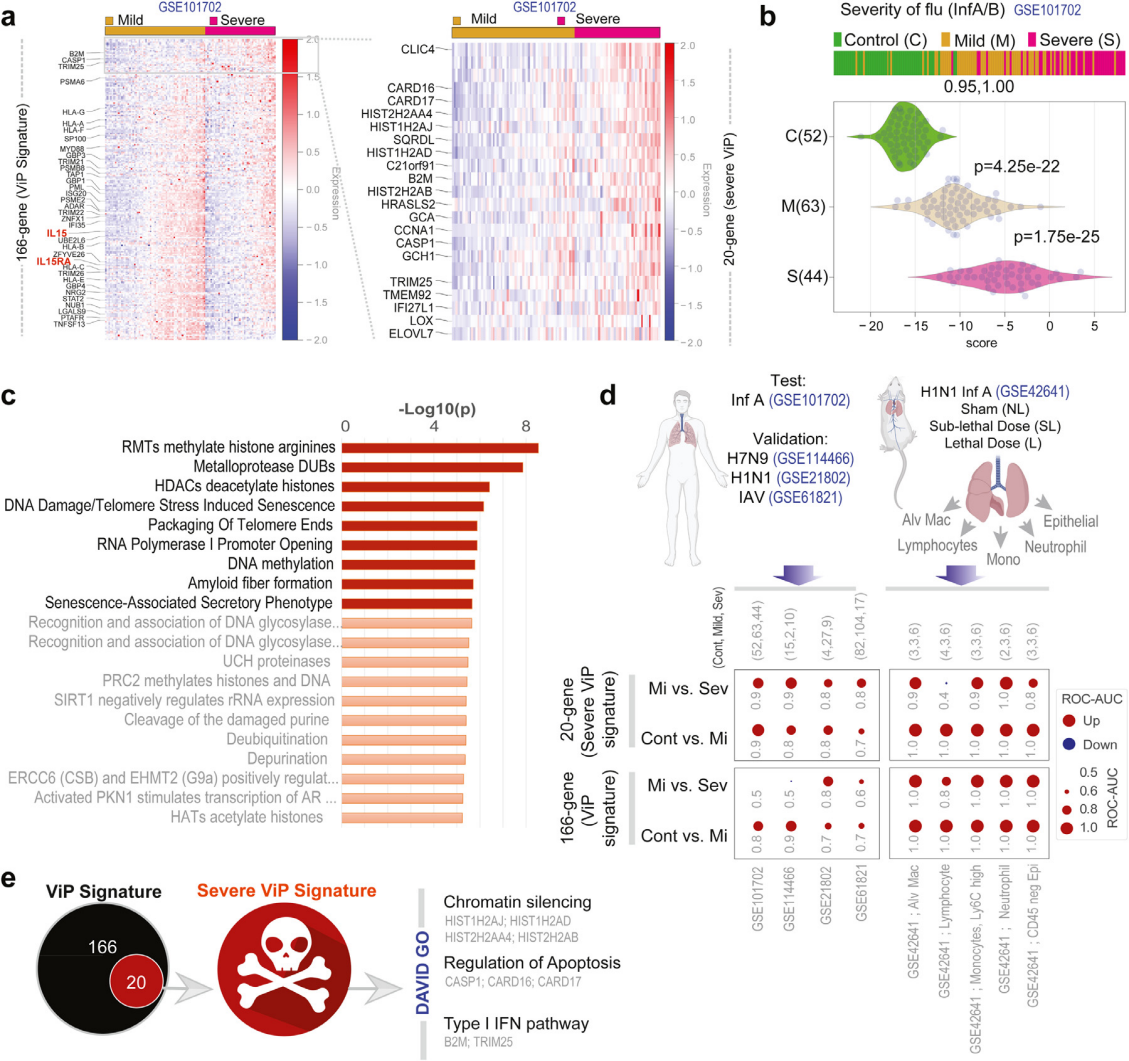
Identification of a ‘severe ViP’ signature. **(a)** Heatmap of the 166 genes on a dataset (GSE101702) annotated with varying severity of infection (healthy controls, 52; mild, 63; severe, 44). Genes are ranked based on their strength of association with severity (T-test between mild and severe). Genes that are involved in cytokine signaling in the immune system are highlighted on the left. Heatmap of top 20 selected genes (‘*severe ViP*’ signature) is shown on the right. **(b)** Bar and violin plots display sample rank order (i.e., classification) of patient samples and distribution of the 20-gene ‘*severe ViP*’ signature in the test dataset (GSE101702). ROC-AUC values of mild and severe cases are shown below the bar plot. **(c)** Reactome pathway analysis of 20 genes. **(d)** Bubble plots of ROC-AUC values (radius of circles are based on the ROC-AUC) demonstrating the direction of gene regulation (Up, red; Down, blue) for the classification based on the 20-gene severe *ViP* signature (top) and 166-gene *ViP* signature (bottom) in the test dataset (GSE101702), three more human datasets (H7N9, GSE114466; H1N1, GSE21802; IAV/H3N1 and others, GSE61821) and one mouse dataset (H1N1 Inf A, GSE42641). For each gene signature, ROC-AUC of controls vs Mild and Mild vs Severe are shown in top and bottom rows, respectively. In the mouse dataset (GSE42641) host response to lethal (L) and sublethal (SL) infection with H1N1 virus were assessed in five different lung cell types: Alv Mac, Lymphocytes, Monocytes, Neutrophil, Epithelial cells. Number of controls, mild and severe cases are shown at the top. **(e)** Summary of the 20-gene severe *ViP* signature and pathway analysis by DAVID GO (https://david.ncifcrf.gov/).

To determine what constitutes ‘severe/fatal’ disease, we rank-ordered the 166 genes within the *ViP* signature for their ability to classify Influenza A/B-infected adult patients by clinical severity [62,63] (*n* = 154; Fig. 4**a**). Severe disease was defined as intubation and mechanical ventilation due to poor oxygenation and/or death. A set of top 20 genes (Fig. 4**a; Table S2, 3–1; Table S4**) was sufficient to classify healthy controls from infected patients (ROC-AUC = 1.00) as well as distinguish mild from severe disease with reasonable accuracy (ROC-AUC = 0.95) in the test cohort (Fig. 4**b**). Reactome pathway analyses revealed that compared to the *ViP* signature, the ‘severity’-related 20-gene cluster enriches a completely different set of cellular processes, i.e., DNA damage (especially induction of genes that are critical for base excision repair; BER), stress-induced senescence, neutrophil degranulation and changes in cell cycle (Figs. 4**c, S2**). We validated this signature side-by-side with the 166-gene *ViP* signature in three human datasets that included samples from mild vs. severe disease during the avian (H7N9), IAV (H3N1 and others) and the swine (H1N1) flu viral pandemics (Fig. 4**d,** *left*). Both the 166-gene *ViP* signature and the 20-gene severity signature performed similarly when it came to classifying control vs. mild disease, but the latter performed significantly better in classifying mild vs. severe disease and did so consistently in both validation datasets (ROC-AUC ranging from 0.8 to 0.9; Fig. 4**d,** *left*).

The severity signature performed well also in a large murine lung dataset in which mice were intranasally infected with non-lethal (NL, control), sub-lethal (SL, mild) and lethal (L, severe) doses of two different strains of H1N1 virus A; the Texas/36/91 (Tx91), which is non-lethal in C57Bl/6 mice and causes transient morbidity and compared against those infected with sublethal and lethal doses of the highly pathogenic Puerto Rico/8/34 (PR8), which causes ARDS and death in less than a week [64]. Harvested lungs were sorted into five different prospectively isolated cell subpopulations and analyzed by microarray (Fig. 4**d,** *right*): alveolar macrophages, lymphocytes (BC, TC, NK), Ly6Chi mononuclear myeloid cells, neutrophils, CD45neg pulmonary epithelial cells. The 166-gene *ViP* signature distinguished the control vs. mild samples perfectly in all five cell types (ROC-AUC = 1.00; Fig. 4**d,** *right*). The classification accuracy of the 20-gene severity signature, however, was most prominent in neutrophils (ROC-AUC = 1.00), followed by monocytes and macrophages (ROC-AUC = 0.9), and then epithelial cells (ROC-AUC = 0.8), but failed in lymphocytes. These findings suggest that the cells of the innate immune system are the primary contributors of disease severity.

We conclude that the 166-gene *ViP* signature that was initially built using *in vitro* infection datasets also detects the host immune response (‘cytokine storm’) in the complex *in vivo* systems; in the *in vivo* context, the response may be triggered by direct viral damage to the lung epithelium but is likely to be propagated by feed-forward dysregulated immune response, both innate and adaptive. Surprisingly, this 166-gene *ViP* signature was not associated with disease severity; instead, severity-associated 20 genes that regulate stress and senescence-associated repression of protein expression and DNA damage (Fig. 4**c**). DAVID GO analyses on the 20-gene signature indicated that 3 biological processes, e.g., transcriptional repression, apoptosis, and intermediates within the type I IFN (IFNγ signaling) pathway (Fig. 4**e**) indicative of cellular distress, senescence/aging and death are the determinants of severity/fatality.

### 5.5 The *ViP* signatures are induced in the lung epithelial and immune cells in COVID-19

**Fig. 5:**
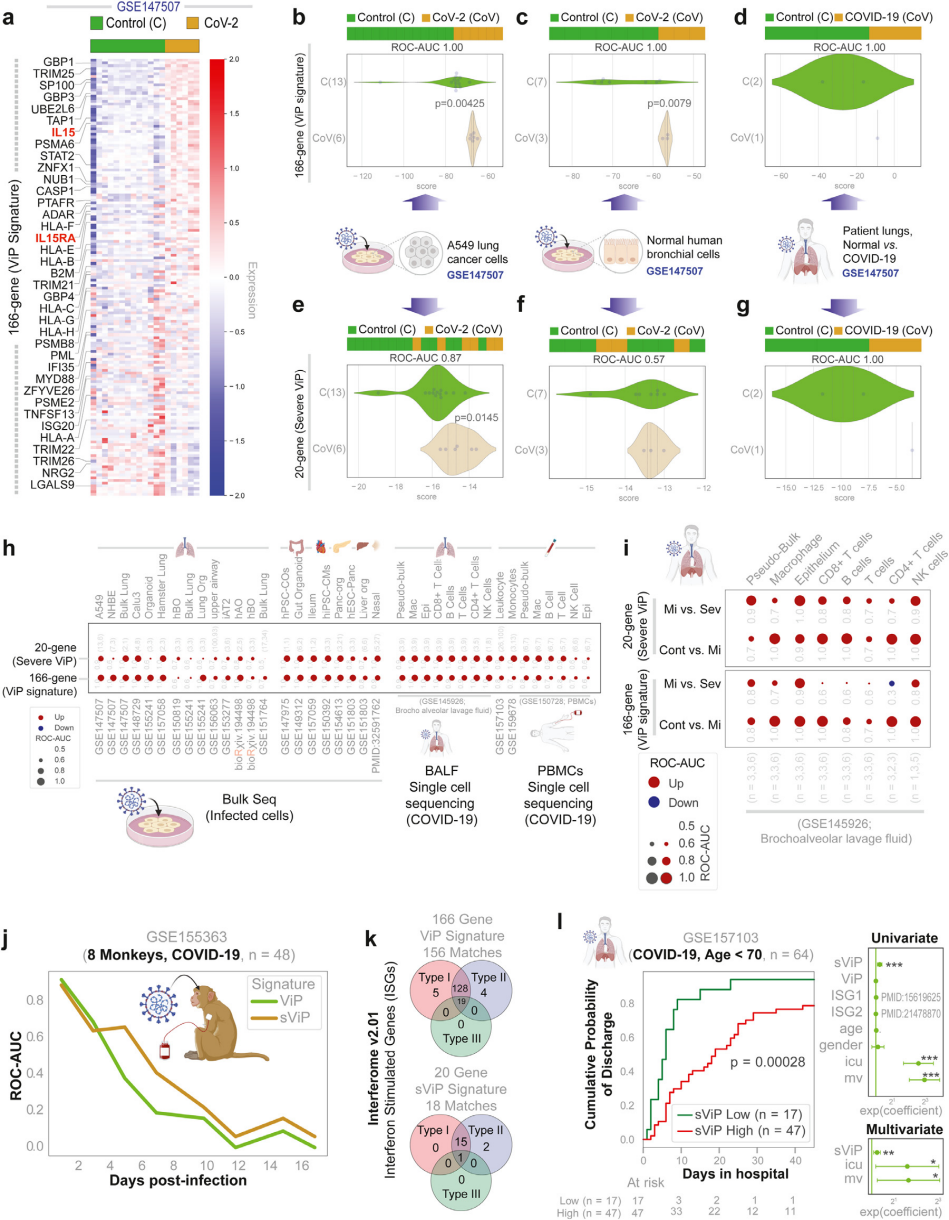
The *ViP* signatures define and measure the host immune response in COVID-19. **(a)** Heatmap of 166 genes in COVID-19 (GSE147507) dataset ranked by genes up-regulated in COVID-19 infected samples. Genes that are involved in cytokine signaling in the immune system are highlighted on the left. **(b–g)** Bar and violin plots displaying sample rank order (i.e., classification) and distribution of gene signature scores of COVID-19 (GSE147507) infected (CoV) and uninfected controls (C) in A549 (13 C, 6 CoV; **b, e**), normal human bronchial epithelial cells (NHBE, 7 C, 3 CoV; **c, f**), and patient lung autopsies (2 Normal, 1 CoV; **d, g**) based on 166-gene (**b–d**) and 20-gene *ViP* signatures (**e–g**). **(h)** Bubble plots of ROC-AUC values (radius of circles are based on the ROC-AUC) demonstrating the direction of gene regulation (Up, red; Down, blue) for the classification based on the 20 gene-severe ViP signature (top) and 166 gene *ViP* signature (bottom) in multiple independent datasets. **(i)** Bubble plots like panel H showing ROC-AUC of controls vs Mild and Mild vs Severe that are shown in the top and bottom rows, respectively, for each gene signature in the COVID-19 single-cell datasets (GSE145926). Dataset is analyzed as a ‘pseudo-bulk’ of all cells or after selecting individual cell types using marker genes specifically expressed in these cell types. **(j)***ViP* and *severe ViP (sViP)* signature-based classification of blood samples (GSE155363) before and up to 17 days after COVID-19 infections in 8 monkeys using ROC-AUC measurements. **(k)** Interferon stimulated genes (ISGs) are annotated in the *ViP* and *sViP* gene lists using Interferome v2.01 web application and displayed as Venn diagrams showing the number of genes regulated by one or more IFN types (Type I, II or III). **(l)** Hospital-free days analysis (45 days followup) of COVID-19 patients (GSE157103) limited to less than 70 years old using *sViP* signature (low and high group) is displayed as Kaplan-Meier estimates (left) of cumulative probability of discharge and its relationship with days in hospital. Cox-proportional hazard univariate analysis (right; *top*) of *sViP* (high vs low) is compared to *ViP* signature, Interferon Stimulated Gene-signatures (ISG1^6^^6^ and ISG2^6^^5^), age, gender, ICU admission (icu) and mechanical ventilation (mv). Multivariate Cox-proportional hazard analysis (right; *bottom*) compares the variables that are significant in univariate settings, i.e., *sViP*, ICU admission (icu) and mechanical ventilation (mv).

We next tested the ability of the *ViP* signatures to distinguish between SARS-CoV-2-infected samples and uninfected controls in 3 independent datasets, 2 of which were datasets generated from cells infected *in vitro* (Fig. 5**a–c**) and one that was generated from lung samples from a fatal case of COVID-19 (Fig. 5**d**). The signature perfectly classified infected from uninfected samples in them all (ROC-AUC 1.00; Fig. 5**a, b, d**); of the 166 genes, both IL15 and IL15RA were notably elevated in infected samples (Fig. 5**a**). The 20-gene signature performed reasonably well in distinguishing infected from uninfected A549 cells (ROC-AUC = 0.87; Fig. 5**e**), and the healthy from the COVID-19 lung sample (ROC-AUC = 1.00; Fig. 5**g**), but not in airway cells (bronchial; ROC-AUC = 0.57; Fig. 5**f**). In fact, the 166-gene and 20-gene signatures perfectly classified infected vs. uninfected samples in all *in vitro* cellular models of CoV-2 infection, regardless of the tissue/organ (Fig. 5**h***; left, middle*). The signatures performed nearly perfectly (ROC-AUC = 0.90 - 1.00; Fig. 5**h**, *right*) across all lung cell types from COVID-19 infected patients analyzed by single-cell sequencing.

We next tested the ability of these signatures to distinguish mild vs. fatal COVID-19 in single-cell sequencing datasets from patient-derived lung samples (Fig. 5**i**). The 166-gene signature was able to distinguish control vs. mild infection most effectively in macrophages, airway epithelium, CD4+ T cells and NK cells (Fig. 5**i**, *lower panel, lower row*) and mild vs. severe disease in the epithelium and in NK cells (Fig. 5**i**, *lower panel, upper row*). The 20-gene signature not only performed well in classifying control vs. mild infection in the same 4 cell types as above but also in B cells and CD8+ T cells (Fig. 5**i**, *upper panel, lower row*). However, the 20-gene severity signature continued to perform most optimally in the epithelium (ROC-AUC = 1.00) and in NK cells (Fig. 5**i**, *upper panel, upper row*). The signatures were also rapidly induced in monkeys challenged with SARS-CoV-2, and gradually suppressed during convalescence after 17 days (Fig. 5**j**).

Because the *ViP* signature is comprised of IFN-signaling pathways and presumably IFN-stimulated genes (ISGs), we asked if the *ViP/severe-ViP* signatures offer any additional advantage beyond ISGs. Using Interferome v2.01 (http://www.interferome.org) we first confirmed that 155/166 genes in *ViP* signature and 18/20 genes in *severe-ViP* signatures were genes that are likely to be regulated by IFN signaling (Fig. 5**k**). Surprisingly, despite such high degree of pathway overlap with ISGs, the *severe-ViP* signature (*sViP*) was able to prognosticate outcome (hospital-free days) in a cohort of patients with COVID-19 (Fig. 5**l**; *left*). When compared head-to-head in an univariate analysis using Cox proportional hazards regression model, the prognostic effect of the *severe-ViP* signature emerged as superior to two different sets of previously published ISGs [65,66] in their ability to prognosticate hospital-free days (Fig. 5**l**; top right). Three factors emerged as determinants of longer hospital stays: (i) the ICU admission status, (ii) need for mechanical ventilation and (iii) induction of the *severe-ViP* signature. A multivariate analysis using Cox proportional hazards regression model suggested that these three factors may be independent covariates of poor outcome (Fig. 5**l**; bottom right).

Together, these findings show that the 166-gene *ViP* signature seen in other respiratory viral pandemics is conserved also in COVID-19. The cytokine storm (166-genes, which included IL15/IL15RA; **Table S2**) was induced in multiple cell types; however, the 20-gene *ViP* signature of disease severity and fatality was most prominently induced in two cell types: (i) the airway epithelial cells, known producers of IL15 after viral infections [67,68] and (ii) the NK cells which are known targets of physiologic as well as overzealous IL15 response [69,70]. Findings also show that despite the ISG-like makeup of the *ViP* signatures, there are key components within the signature that is able to detect disease severity.

### 5.6 Viral infection and IL15 induce, and Flu vaccine attenuates the *ViP* signatures in NK cells

**Fig. 6:**
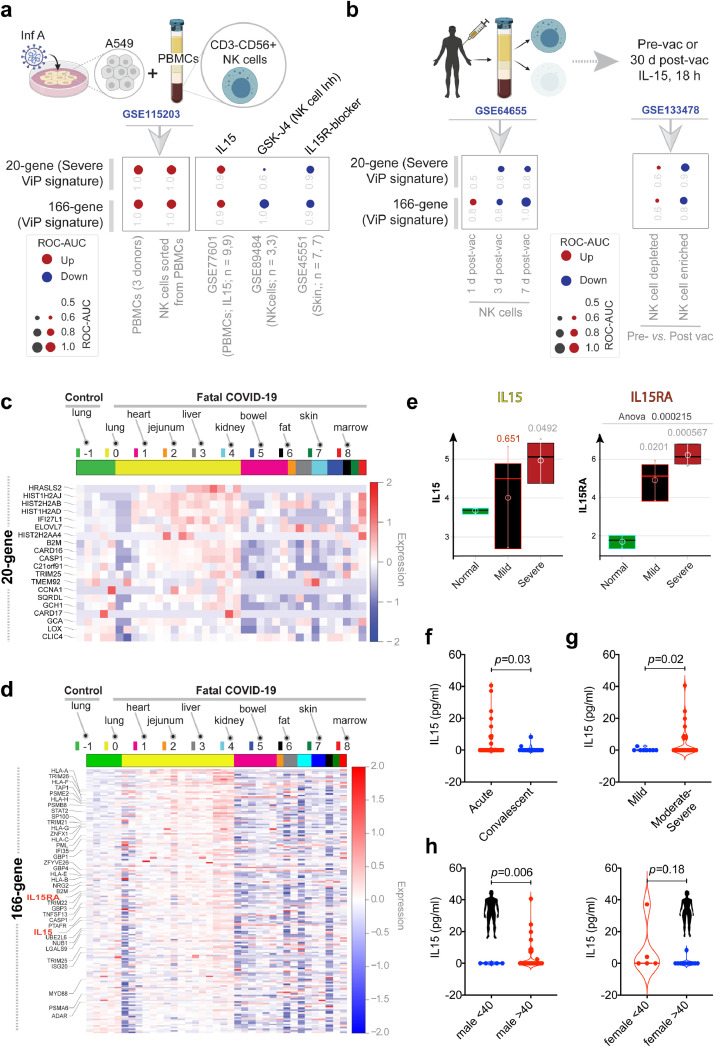
*ViP* signatures reveal an interplay between IL15-storm and NK cell dysfunction in fatal COVID-19. **(a)** Bubble plots of ROC-AUC values (radius of circles are based on the ROC-AUC) demonstrating the direction of gene regulation (Up, red; Down, blue) for the classification based on the 20 gene severe *ViP* signature (top) and 166 gene *ViP* signature (bottom) in following datasets. RNASeq data (GSE115203) from PBMCs and sorted NK cells from PBMCs incubated with uninfected A549 cells for 12 hrs compared to infected A549 cells. PBMCs treated with IL15 compared to IL2 (GSE77601). RNASeq analysis of NK cells (GSE89484) treated with GSK-J4 compared to DMSO. Skin tissue in mice (GSE45551) is treated with anti-IL15RB antibody compared to PBS. **(b)** RNASeq data of NK cells isolated from two donors prior to vaccination compared (left) to days 1, 3, and 7 post-TIV vaccination like panel A. RNASeq data of NK enriched and NK depleted PBMCs from healthy donors compared to 30 day post-vaccination like panel A. **(c, d)** Heatmap of 20-gene (panel **c**) and 166-gene (panel **d**) *ViP* signatures in tissues collected during rapid autopsies on patients who succumbed to COVID-19. Genes are ranked according to the strength of differential expression (T-test) in lung tissue between normal and infected tissue. **(e)** Box plots of IL15 and IL15RA in samples from varying severity of COVID-19. **(f-h)** Violin plots show levels of plasma IL15 in COVID-19 patients stratified by disease acquity (F), by clinical severity (G) and by gender and age (H). Welch's two sample unpaired *t*-test is performed to compute the p values. See also **Table S5** for patient metadata.

NK cells are known to lyse influenza virus-infected cells by direct cytotoxicity and antibody-dependent cellular cytotoxicity (ADCC); enhancing such NK cell function has been shown to control influenza virus infections [71]. Clearance of other viruses (HIV-1, other retroviruses, etc.) and cancer immunotherapies also leverage such NK cell-dependent ADCC [72,73]. Because IL15, the only cytokine within the *ViP* signature, is critical for NK cell activation and exhaustion [69,70], we analyzed datasets from NK cells exposed to virus-infected epithelial cells. More specifically, a transcriptomic dataset (GSE115203) [74] generated from co-culture studies of human PBMCs (3 donors) with influenza (H1N1 Puerto Rico/08/1934)-infected airway epithelial cells (A549) was analyzed (Fig. 6**a**; *top*). PBMCs (from co-culture), or NK cells FACS-sorted from the PBMC were then analyzed by RNA Seq, and the study had confirmed NK cell ADCC responses were durably induced in this assay *via* type I IFN release from PBMCs. We found that both the 166- and 20-gene *ViP* signatures were induced in PBMCs and in NK cells sorted from the PBMCs (Fig. 6**a**; *bottom left*), indicating that NK cells in these co-culture models were sufficient to capture the observed host immune response in patients with COVID-19.

To test the role of IL15 in the induction of *ViP* signatures, we leveraged three datasets—one that used recombinant IL15 (PBMCs; GSE77601), another that used anti-IL1Rβ mAb (mouse skin biopsies; GSE45551) [75], and a third study using the prototypic H3K27 demethylase inhibitor, GSK-J4; the latter was shown to inhibit NK cell effector cytokines in response to IL15 without impacting its cytotoxic killing activities (human, NK cells; GSE89484) [76]. Both *ViP* signatures were stimulated by IL15 but attenuated in the two other datasets where IL15’s actions were blocked pharmacologically (Fig. 6**a;** *right*). These findings indicate that IL15 could be necessary and sufficient to induce the *ViP* signatures.

Because two independent studies [77,78] (one of them [78] being a preprint) recently showed that those vaccinated against influenza have lower odds of requiring intensive care, invasive ventilation and/or dying, we analyzed two transcriptomic datasets (GSE64655 [79] and GSE133478 [80]) in which PBMCs from subjects vaccinated with seasonal trivalent or quadrivalent influenza vaccine (TIV/QIV) were collected and analyzed for NK cell activation. The first study showed that the NK cells continued to demonstrate progressive attenuation of both the 166- and 20-gene signatures rapidly within 7 days (Fig. 6**b**, *left*). The second study, in which the NK cell-enriched and depleted fractions collected pre- and post (30 d)-vaccination were tested for their response to re-stimulation with IL15 (low dose, 0.75 ng/ml, 18 h); such stimulation is known to enhance NK cell activity [[81], [82], [83]] and promote viral clearance [[84], [85], [86]]. Both *ViP* signatures were attenuated post-vaccination in NK cell-enriched fractions, but not in depleted fractions (Fig. 6**b**, *right*). Because such post-vaccination attenuation happened in the setting of experimentally confirmed [80] enhancement of overall NK cell response, we conclude that attenuation of *ViP* signatures among recipients of TIV could continue to offer protection during re-challenge. Because such protection is seen in NK-cell enriched, but not depleted fractions, we conclude that the protection is mediated primarily *via* the preservation of functional NK cells.

### 5.7 An IL15-storm originating in the lung alveoli determines the severity of COVID-19

We next analyzed the *ViP* signatures in transcriptomic datasets generated from multiple organs at autopsy. Both the 166- and 20-gene *ViP* signatures were predominantly enhanced in one organ, the lungs (Fig. 6**c,d**); and IL15/IL15RA were also elevated in the lungs (Fig. 6**d**). These findings indicate that the 20- and 166-gene signatures go together and suggest a plausible cause and effect relationship. For instance, severity-related cellular events (such as epithelial and NK cell senescence) occur in the milieu of the organ that mounts the highest IL15-predominant cytokine response, i.e., lungs. We also found that IL15 and its receptor IL15RA were significantly increased in severe COVID-19 lungs (Fig. 6**e**). These findings predict that an overzealous IL15-predominant cytokine response is the most consistent finding in the most severe cases of COVID-19 and that the lung epithelium is the likely source of such a storm.

**Fig. 7:**
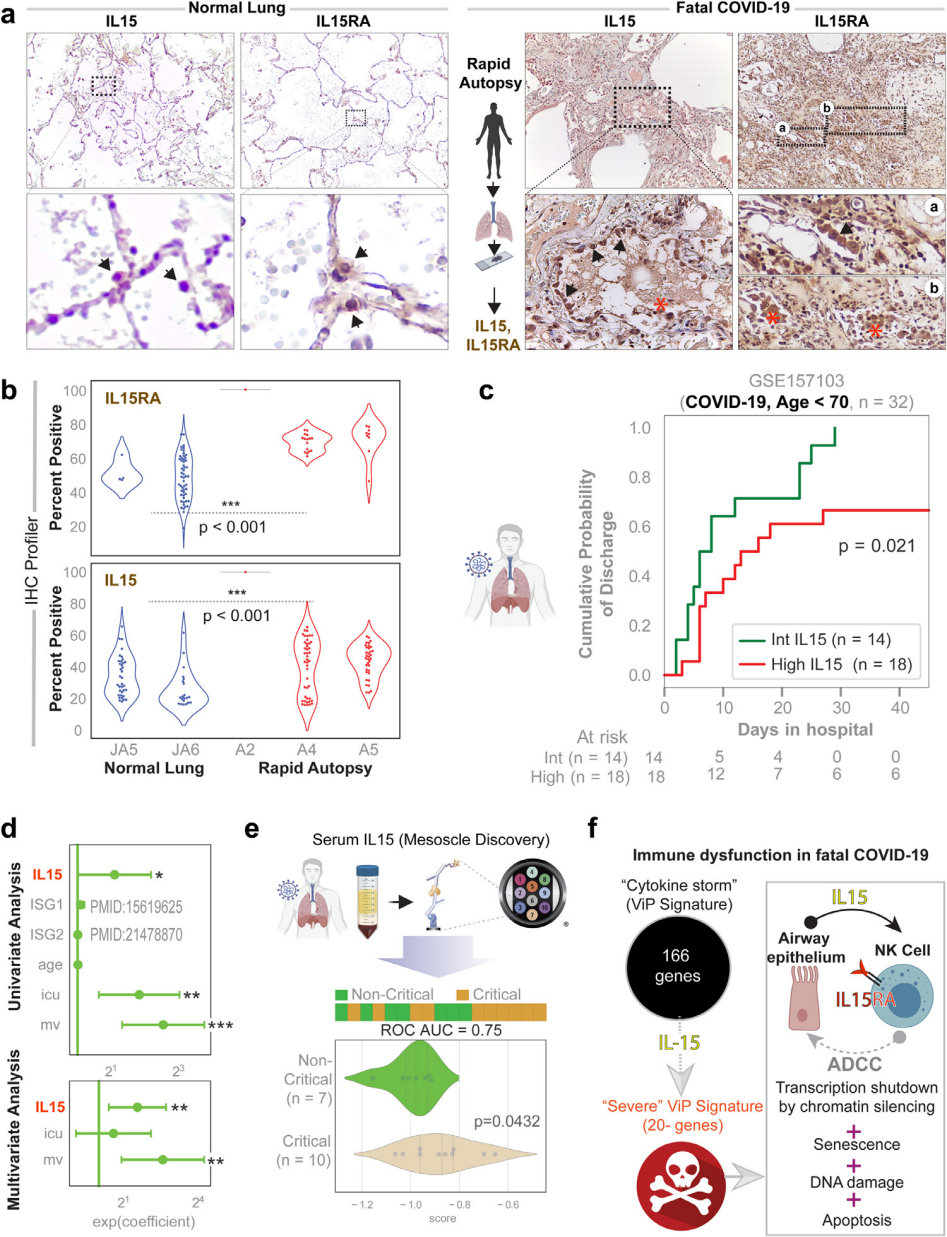
Lung alveolar cells contribute to the IL15 storm in fatal COVID-19. (**a**) Normal lung tissue obtained during surgical resection (*left*) or lung tissue obtained during autopsy studies on COVID-19 patients (*right*) were stained for IL15 and IL15RA. Representative images are shown. Mag = 10X. (**b**) Violin plots display the intensity of staining for IL15RA (top) and IL15 (bottom), as determined by IHC profiler. (**c**) Hospital-free days analysis (45 days followup) of COVID-19 patients (GSE157103) limited to males less than 70 years old using the abundance of IL15 transcripts (intermediate and high groups) is displayed as Kaplan-Meier estimates (left) of cumulative probability of discharge and its relationship with days in hospital. (**d**) Cox-proportional hazard univariate analysis (right; *top*) of *sViP* (high vs low) is compared to *ViP* signature, Interferon Stimulated Gene-signatures (ISG1, PMID:15619625; ISG2, PMID:21478870), age, gender, ICU admission (icu) and mechanical ventilation (mv). Multivariate Cox-proportional hazard analysis (right; *bottom*) compares the variables that are significant in univariate settings, i.e., *sViP*, ICU admission (icu) and mechanical ventilation (mv). (**e**) *Top*: Schematic displays the workflow for patient blood collection and assessment of IL15 levels by mesoscale. *Bottom*: Bar (top) and violin (bottom) plots for the levels of IL15 cytokine (score = *Z* score of the log reduced mesoscale concentration data). ROC AUC numbers indicate the strength of classification between patients with critical/fatal disease course vs. those with non-critical infection. (**f**) Summary of IL15 signaling and the hypothetical role of NK cells in the severity of COVID-19 infections.

These predictions were validated in a cohort of symptomatic COVID-19 patients who presented to the UC San Diego Medical Center with varying disease severity, ranging from mild to fatal (see **Table S5**). Plasma ELISA studies revealed that IL15 levels were significantly elevated during the acute compared to the convalescent visit (Fig. 6**f**), and in whom the clinical presentation was moderate-to-severe compared to those with mild disease (Fig. 6**g**). A sub-group analysis confirmed that while gender or age did not have a significant impact on plasma IL15 levels independently, the aged male (> 40 y) cohort had a significantly higher IL15 level than the young males (Fig. 6**h**; *left*). No such pattern was noted among females. These findings are consistent with the fact that the gender gap in COVID-19-related deaths widens markedly with age [87]. Lungs collected during autopsies from patients who succumbed to COVID-19 (see **Table S6**) further confirmed that lung epithelial cells, especially the alveolar type II pneumocytes and alveolar immune cell infiltrates express high levels of IL15 and its receptor, IL15RA (Fig. 7**a, b**).

Finally, in a cohort of patients with COVID-19, high levels of IL15 transcript carried a poor prognosis (lower probability of discharge from the hospital; Fig. 7**c**). An univariate analysis using Cox proportional hazards regression model showed that the prognostic effect of high-IL15 was superior to ISGs [65,66] (Fig. 7**d**; left), as we observed previously for ViP signatures (Fig. 5**L**). A multivariate analysis using Cox proportional hazards regression model suggested that need for mechanical ventilation and IL15 induction may be independent covariates of poor outcome (Fig. 7**d**; right). That the serum IL15 levels track disease severity was validated in a cohort of patients presenting to our institution with a diagnosis of COVID-19; critical/fatal disease was associated with significant elevation of the cytokine (Fig. 7**e**).

Taken together, these findings support the following model of the immunopathogenesis of COVID-19 (Fig. 7**f**): Airway epithelial cells and cells of the myeloid lineage and other immune cells are the primary source of the 166-gene cytokine storm, of which, IL15 is a component. It is possible, that the primary target of IL15, i.e., NK cells, when exposed to this storm for a prolonged period undergo damage, stress-induced senescence and apoptosis. Our model is consistent with prior studies showing that the airway epithelial cells (especially bronchial) constitutively express the IL15 and IL15RA/B genes and that viral infections and IFNγ can induce the synthesis and secretion of IL15 [68], and that prolonged and excessive stimulation with IL15 is known to induce NK cell exhaustion [69,70]. These findings are consistent with the emerging reports that NK cells are significantly exhausted and reduced in cases of severe COVID-19 infection [88,89] and that such reduction was seen as early as 3–6 days after the onset of symptoms [90]. We conclude that fatal COVID-19 is characterized by a paradoxical immune response, i.e., suppression of epithelial and NK cell functions (immunosuppression) in the setting of a cytokine storm (overzealous immune response).

### 5.8 The *ViP* signatures formulate therapeutic goals, track treatment efficacy

**Fig. 8:**
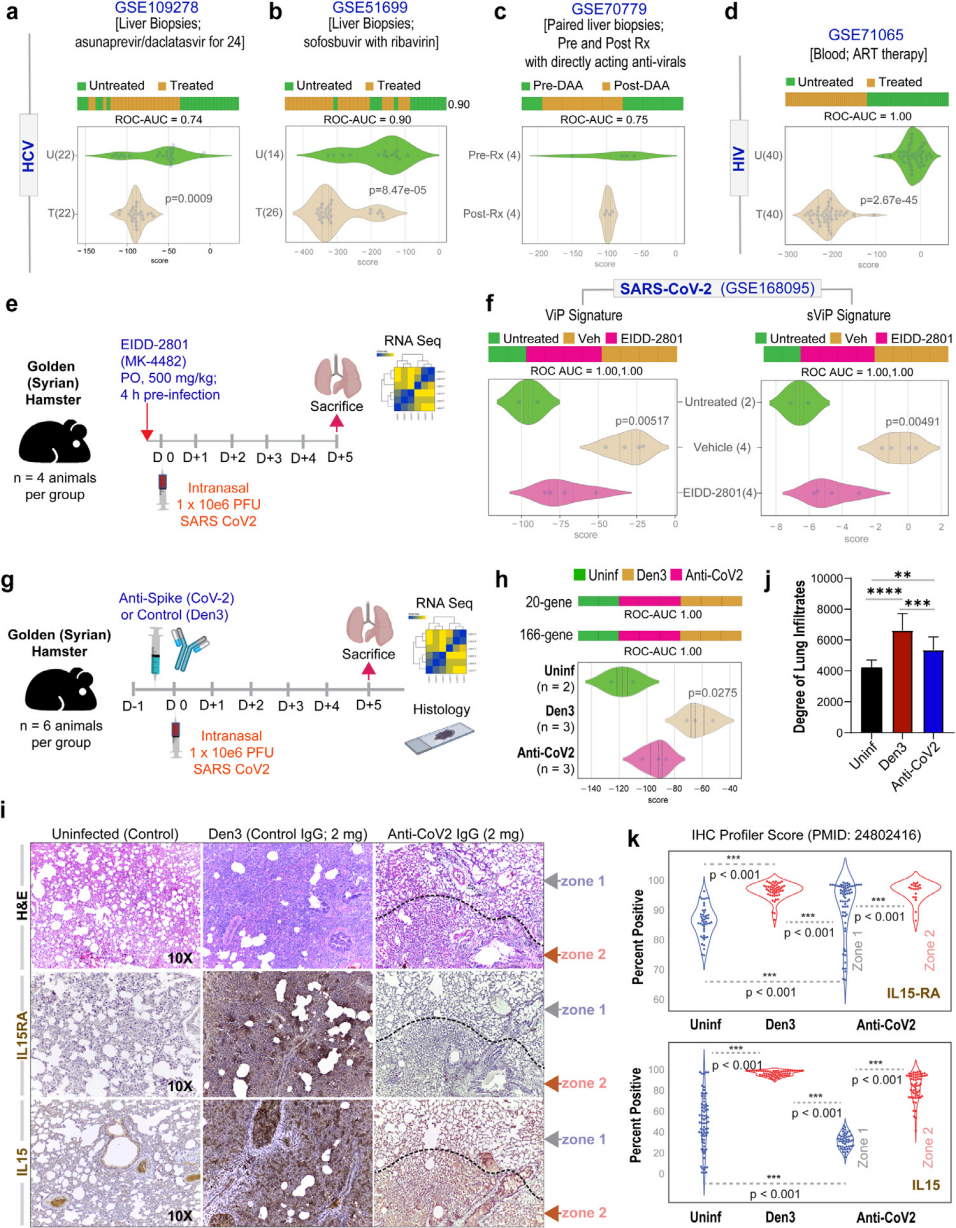
Validation of *ViP* signature-guided therapeutic goals. **(a-c)** The 166-gene *ViP* signature-was used to classify liver biopsies from HCV-infected patients treated or not with directly acting anti-viral agents. ROC-AUC values are shown below each bar plot unless otherwise stated. **(d)** 166-gene *ViP* signature-based classification of blood samples from HIV-infected patients treated with anti-retroviral therapy (ART). **(e)** The compound EIDD-2801 (MK-4482; 500 mg/kg) or vehicle (*Veh*) was administered at indicated doses to Golden Syrian hamsters 4 h prior to intranasal infection with SARS-CoV-2. Hamsters were sacrificed on day 5 and lungs we analyzed by RNA sequencing. **(f)** Bar (top) and violin (bottom) plots using the *ViP* (left) or *sViP* (right) signature-based classification of lung samples from hamsters in E and uninfected controls. **(g)** Schematic showing the experimental design for validating the *ViP* signatures as useful tools to assess therapeutic efficacy. *Uninf*, uninfected; *Den3 and Anti-CoV-2* indicate SARS-CoV-2 challenged groups that received either a control mAb or the clone CC12.2 of anti-CoV-2 IgG, respectively. (**h**) Bar (*top*) and violin (*bottom*) plots display the 166- and 20-gene *ViP* signatures in the uninfected and the SARS-CoV-2 challenged groups, treated with control or anti-CoV-2 IgG. (**i-k**) Lungs harvested from the 3 groups of hamsters were analyzed by H&E and IHC. Representative images are shown in I. Mag = 10X. Bar graphs in J display the abundance of cellularity and infiltrates in the lungs of the 3 groups, as determined by ImageJ. Violin plots in K display the intensity of staining for IL15RA (*top*) and IL15 (*bottom*), as determined by IHC profiler.

Previously we showed that the attenuation of the *ViP* signature was ‘associated’ with the acquisition of natural convalescence in several respiratory viral pandemics (Fig. 2**f–h**); we now asked if they could serve as a readout of therapeutic efficacy. We analyzed interventional studies in the setting of other viral infections that shared the *ViP* signature, i.e., HCV, HIV, Zika and Ebola (Fig. 3**h; Fig. S1; Table S3**). The 166-gene *ViP* signature classified HCV-infected liver biopsies treated or not with directly acting anti-viral agents (DAAs) (Fig. 8**a–c**) and HIV-infected samples treated or not with *a*nti-*r*etroviral *t*herapeutics (ART; ROC-AUC = 1.00; Fig. 8**d**) with sufficient accuracy. In the case of Ebola, the *ViP* signature was somewhat effective in classifying crisis (i.e., acute) from convalescent PBMC samples (ROC AUC 0.64; **Fig. S4a, top**), and previously described anti-Ebola therapeutic strategies (Topoisomerase depletion with siRNA [91] inhibited the signature in Ebola-infected alveolar epithelial cells (siTop; ROC AUC 1.00; **Fig. S4a, bottom**) [91]. Finally, the *ViP* signature was accentuated in Zika infected human cortical neural progenitor cells (**Fig. S4b**) and was effectively attenuated when these infected samples were treated with two investigational drugs that were found to be effective in inhibiting Zika infection. These findings imply that attenuation of the 166-gene *ViP* signature is a desirable therapeutic goal.

We next sought to determine if the SARS-CoV-2 virus can induce the *ViP* signatures, and whether the signatures can track therapeutic response. We tested two therapeutic approaches. The first approach was the use of N-hydroxycytidine, the parent of the prodrug MK-4482 (Molnupiravir, EIDD-2801) which has not only proven as a potent and selective oral antiviral nucleoside analogue in mice, guinea pigs, ferrets and human airway epithelium organoids [[92], [93], [94], [95], [96]], but also showing promise in Phase IIa trials in the treatment of COVID-19 patients (NCT04405570). We analyzed by RNA seq the lungs from SARS-CoV-2-challenged golden Syrian hamsters who were pre-treated either with this drug or vehicle control (see study protocol in Fig. 8**e**). Both 166- and 20-gene *ViP* signatures were induced in the vehicle-treated arm, and effectively suppressed in the drug-treated arm to levels close to uninfected controls (GSE168095; Fig. 8**f**). The second approach was the use of SARS-CoV-2-neutralizing antibodies whose design was inspired by monoclonal antibodies (mAbs) isolated from convalescent donors [14]. A specific isotype of this antibody, which binds to the receptor-binding domain (RBD-A) of SARS-CoV-2 spike protein in a fashion that precludes binding to host ACE2, was demonstrated as effective in preventing infection and weight loss symptoms, in cell-based and *in vivo* hamster models of infection, respectively. We observed that SARS-CoV-2-challenged hamsters that were pre-treated with anti-CoV-2 antibody, but not the control Den3 antibody (see Fig. 8**g** for study protocol) had 3 key findings: (i) they suppressed both the 166- and 20-*ViP* signatures that were otherwise induced in the infected lungs (GSE157058; Fig. 8**h**); (ii) their lungs were protected from overwhelming immune cell infiltration and obliteration of alveolar space (Fig. 8**i, j**); (iii) expression of IL15 and IL15 receptor was significantly reduced compared to what was observed in the infected lungs (Fig. 8**i, k**).

These results validate the ACE-centric computational approach for identifying the *ViP* signatures, i.e., when ACE2•virus engagement was disrupted using antibodies, or reduced using directly acting anti-virals that prevent viral replication using Molnupiravir, the signatures were suppressed. The findings also indicate that the reversal of the signature and the IL15 storm could be used as a readout of therapeutic efficacy.

## 6 Conclusion

The major and unexpected finding in this work is that all viral pandemics (regardless of their acuity, causative virus, case fatality rates and clinical presentation) share a common fundamental host immune response. Summarized below are our three major findings.

*First*, we defined an invariant 166-gene host response –the so-called “cytokine storm”– that is surprisingly conserved among all viral pandemics. Such conservation was unexpected because the use of ACE2 as a ‘seed’ gene was rationalized because SARS-CoV-2 enters the host cell by engaging the host ACE2 receptor via its receptor-binding domain (RBD). Host receptor recognition for cell entry is, however, not specified by the CoV genus classification. MERS-CoV is a member of the beta(B)-CoV genus but does not recognize the ACE2 receptor [97], and yet, were found to induce the *ViP* signatures. By contrast, the alpha(A)-CoV HCoV-NL63 does recognize the ACE2 receptor [30,98]. Similarly, the IAV, HIV or Ebola viruses also do not recognize the ACE2 receptor, and yet, induce the *ViP* signatures. This suggests that while ACE2 may be the entry site for SARS-CoV-2 and a few other CoVs, it is a prominently upregulated gene during host response to other viral infections. As a key regulator of the renin-angiotensin system (RAS), ACE2 expression is increased in the setting of multiple stressors, including non-CoV-2 infections. For example, IAV, H7N9 and rhinoviruses amplify the expression of ACE2 in the distal lung [[99], [100], [101]]. ACE2 activity is also induced in bacterial lung infections [35]. In fact, ACE2 protects against acute lung injury in several animal models of ARDS [102]. In fact, when we carried out additional analyses to understand how the downstream results will change if a different seed gene was used, we found that the signature was robust to such changes. For example, if we choose seed genes from the 166 genes, more than 75% of the genes matched more than 75% with the 166-gene signature. When using an immune-related gene as ‘seed’, e.g., LMO2 matched 92% (130/141) with the ACE2-centric 166 genes. Similarly, other relevant immune genes such as TRIM26, IL15RA, HLA-E, HLA-H, HLA-B, TLR2, and TLR3 as seed genes individually matched more than 82% with the ACE2-centric 166 genes. This suggests that a majority of the downstream genes would remain regardless of which reasonably relevant immune gene is used as a ‘seed’ gene. Thus, retrospectively, ACE2 is not as specific a ‘seed’ gene for SARS-CoV-2 as was assumed; neither is it specific for other microbes. It is not so surprising that the ACE2-equivalent *ViP* signature is more generalizable as a signature that is induced in respiratory infections”.

*Second,* we define the precise nature of the cytokine storm and pinpoint the IL15 cytokine and its receptor, IL15RA as invariant components. We demonstrate that systemic levels of IL15 track disease severity among patients and that the levels are notably elevated in the aged male (the predisposed age group in COVID-19, as per reports worldwide). Using a combination of single-cell RNA Seq and human lung histology, we also pinpoint the lung epithelial and myeloid cells as the key contributors to the *ViP* signature, and more specifically, IL15/IL15RA. These findings were recently validated in another concurrent publication [103]– multivariate analyses of soluble biomarkers identified that increased IL-15 is independently associated with mortality and that the levels of the cytokine were consistently high throughout the hospitalization in patients who died versus those who recovered.

*Third*, we found that a subset of 20-gene ‘severe’ *ViP* signature, indicative of stress-induced senescence, transcriptional repression, DNA damage and apoptosis is also shared among various viral pandemics. In patients with COVID-19, this signature was seen in lung epithelial and NK cells, which is intriguing because airway epithelial cells is a prominent source and the NK cells are a major target of IL15. Thus, the *ViP* signatures begin to paint a picture of ‘paradoxical immunosuppression’ at the heart of fatal COVID-19, in which, the observed NK cell exhaustion/depletion in severe COVID-19 [88], [89], [90][104] could be a consequence of an overzealous IL15 storm, leading to their senescense and apoptosis.

As for limitations of our study, our choice of computational approach, i.e., BECC, for the initial analysis step in the pipeline entails Boolean analysis instead of linear algebra. While some argue that this approach (i.e. binarization, discretization) improves robustness, others raise valid criticisms that key information may be lost. We argue that all methodologies have strengths and weaknesses; in this case traditional analyses leads to overfitting of data, which lacks reproducibility when applied to other datasets, a Boolean formula filters out the noise in a large-scale diverse data setting, thereby making it more generalizable. However, such generalizability comes at a cost of losing some critical information. Typically, distinguishing the critical information that is lost from the noise is difficult.

In closing, given that the emerging pandemic is still largely a mystery to us in terms of how it picks its victims, the *ViP* signature we define here provides a computational framework for navigation in otherwise uncharted territory. While it is expected that the signature will be more effective and accurate when it is iteratively filtered using emerging COVID-19 datasets, we provide evidence for its usefulness now in formulating therapeutic strategies and rapidly screening for therapeutics. Because the *ViP* signature of host response is seen also in other viral pandemics tested, findings may also be relevant also in navigating management strategies in those pandemics.

## Contributors

D.S., P.G. and S.D conceptualized the study, D.S., P.G., S.D. S.T., M.B., G.D.K., C.T, M.R.F., S.K., A.C., V.C.participated in investigation, methodology and data curation, D.S, G.D.K, S.D. and P.G carried out formal analysis, D.S, S.D and P.G were responsible for funding acquisition, D.S, S.D and P.G administered the project, T.F.R, N.B, D.R.B, J.D., L.C.A, S.C., J.D., S.I.R, V.P., S.A.R, D.M.S provided essential resources (hamster tissues, human serum and tissues, etc.), D.S provided software, D.S, S.D and P.G provided supervision, D.S, G.K and P.G led efforts in data visualization, P.G and D.S led efforts in writing – original draft, and G.K, S.D., P.G and D.S contributed to writing – review & editing. D.S, S.D and P.D have accessed and verified the underlying data. All authors read and approved the final version of the manuscript.

## Declaration of Competing Interest

The authors declare no competing interests.

## Appendix Supplementary materials

Image, application 1

Image, application 2

Image, application 3

Image, application 4

Image, application 5

Image, application 6

Image, application 7

Image, application 8
