# Supplementary material for: AI-guided discovery of the invariant host response to viral pandemics
Source: eBioMedicine. 2021 Jun 11;68:103390. doi: 10.1016/j.ebiom.2021.103390 (PMC8193764; doi:10.1016/j.ebiom.2021.103390)
Supplement: Supplementary file 2 [file mmc2.docx]

**Table S2:**

**1-1: List of genes that are equivalent to ACE2 that are elevated in acute infection and reduced in convalescent SARS-CoV infection (ViP signature)** **[n = 166]**

| ABCD1 | ACE2 | ADAMTSL3 | ADAR | ADPRHL2 | APOBEC3B | APOL4 | APOL6 |
| --- | --- | --- | --- | --- | --- | --- | --- |
| AZI2 | B2M | BCL2L14 | BLZF1 | BRIP1 | C19orf66 | C21orf91 | C3orf38 |
| C6orf62 | CARD16 | CARD17 | CASP1 | CCNA1 | CD274 | CHMP5 | CLIC4 |
| CNP | COL16A1 | CSAG1 | CSAG2 | CXCL16 | CYP21A2 | CYP2J2 | DCLRE1C |
| DHX58 | DTX3L | ELOVL7 | ETV7 | FAM26F | FANCA | FAS | FBXO6 |
| FGD6 | FRMD3 | GBP1 | GBP3 | GBP4 | GCA | GCH1 | GLRX |
| GMPR | GPT2 | GTPBP1 | HDX | HESX1 | HIST1H2AD | HIST1H2AJ | HIST2H2AA4 |
| HIST2H2AB | HLA-A | HLA-B | HLA-C | HLA-E | HLA-F | HLA-G | HLA-H |
| HRASLS2 | HSH2D | HSPB9 | HTR2B | IDO1 | IFI16 | IFI27L1 | IFI35 |
| IFNE | IGFBP4 | IL15 | IL15RA | IL4I1 | ISG20 | KCNE4 | KLHDC7B |
| LAP3 | LGALS3BP | LGALS9 | LGALS9C | LMO2 | LOX | LPAR6 | LYSMD2 |
| MASTL | MMAA | MOV10 | MUC13 | MYD88 | MYH7 | N4BP1 | NAT8 |
| NCOA7 | NMI | NRG2 | NUB1 | NUPR1 | OPTN | PANX1 | PARP12 |
| PARP14 | PARP9 | PDCD1LG2 | PHF11 | PLSCR1 | PML | PNPT1 | PPP2R2A |
| PSMA6 | PSMB8 | PSME2 | PTAFR | PTPRR | RBCK1 | RBMS2 | RNF114 |
| RNF19B | RTP4 | S1PR2 | SAMD9 | SCARB2 | SCO2 | SECTM1 | SHISA5 |
| SLC15A3 | SLC16A1 | SLC25A28 | SLC8A2 | SP100 | SPATS2L | SQRDL | STARD5 |
| STAT2 | TAP1 | TDRD7 | TLR2 | TLR3 | TMEM140 | TMEM62 | TMEM92 |
| TNFSF10 | TNFSF13 | TOR1B | TRAFD1 | TREX1 | TRIM21 | TRIM22 | TRIM25 |
| TRIM26 | TRIM69 | TYMP | UBA6 | UBE2L6 | UNC93B1 | VAMP5 | WARS |
| WDFY1 | ZBP1 | ZFYVE26 | ZNF618 | ZNF620 | ZNFX1 |  |  |

**1-2: Reactome analysis ACE2 equivalent genes that are elevated in acute infection and reduced in convalescent SARS-CoV infection [n = 166]**

| Name | pValue | FDR |
| --- | --- | --- |
| [ER-Phagosome pathway](http://www.reactome.org/PathwayBrowser/#/R-HSA-1236974&DTAB=AN&ANALYSIS=MjAyMDAzMjEwNDQwMzlfNDc3) | 1.1102230246251565e-16 | 5.773159728050814e-15 |
| [Antigen Presentation: Folding, assembly and peptide loading of class I MHC](http://www.reactome.org/PathwayBrowser/#/R-HSA-983170&DTAB=AN&ANALYSIS=MjAyMDAzMjEwNDQwMzlfNDc3) | 1.1102230246251565e-16 | 5.773159728050814e-15 |
| [Interferon alpha/beta signaling](http://www.reactome.org/PathwayBrowser/#/R-HSA-909733&DTAB=AN&ANALYSIS=MjAyMDAzMjEwNDQwMzlfNDc3) | 1.1102230246251565e-16 | 5.773159728050814e-15 |
| [Endosomal/Vacuolar pathway](http://www.reactome.org/PathwayBrowser/#/R-HSA-1236977&DTAB=AN&ANALYSIS=MjAyMDAzMjEwNDQwMzlfNDc3) | 1.1102230246251565e-16 | 5.773159728050814e-15 |
| [Class I MHC mediated antigen processing & presentation](http://www.reactome.org/PathwayBrowser/#/R-HSA-983169&DTAB=AN&ANALYSIS=MjAyMDAzMjEwNDQwMzlfNDc3) | 1.1102230246251565e-16 | 5.773159728050814e-15 |
| [Antigen processing-Cross presentation](http://www.reactome.org/PathwayBrowser/#/R-HSA-1236975&DTAB=AN&ANALYSIS=MjAyMDAzMjEwNDQwMzlfNDc3) | 1.1102230246251565e-16 | 5.773159728050814e-15 |
| [Interferon Signaling](http://www.reactome.org/PathwayBrowser/#/R-HSA-913531&DTAB=AN&ANALYSIS=MjAyMDAzMjEwNDQwMzlfNDc3) | 1.1102230246251565e-16 | 5.773159728050814e-15 |
| [Immunoregulatory interactions between a Lymphoid and a non-Lymphoid cell](http://www.reactome.org/PathwayBrowser/#/R-HSA-198933&DTAB=AN&ANALYSIS=MjAyMDAzMjEwNDQwMzlfNDc3) | 1.1102230246251565e-16 | 5.773159728050814e-15 |
| [Adaptive Immune System](http://www.reactome.org/PathwayBrowser/#/R-HSA-1280218&DTAB=AN&ANALYSIS=MjAyMDAzMjEwNDQwMzlfNDc3) | 1.1102230246251565e-16 | 5.773159728050814e-15 |
| [Immune System](http://www.reactome.org/PathwayBrowser/#/R-HSA-168256&DTAB=AN&ANALYSIS=MjAyMDAzMjEwNDQwMzlfNDc3) | 1.1102230246251565e-16 | 5.773159728050814e-15 |
| [Interferon gamma signaling](http://www.reactome.org/PathwayBrowser/#/R-HSA-877300&DTAB=AN&ANALYSIS=MjAyMDAzMjEwNDQwMzlfNDc3) | 1.1102230246251565e-16 | 5.773159728050814e-15 |
| [Cytokine Signaling in Immune system](http://www.reactome.org/PathwayBrowser/#/R-HSA-1280215&DTAB=AN&ANALYSIS=MjAyMDAzMjEwNDQwMzlfNDc3) | 1.1102230246251565e-16 | 5.773159728050814e-15 |
| [Formation of editosomes by ADAR proteins](http://www.reactome.org/PathwayBrowser/#/R-HSA-77042&DTAB=AN&ANALYSIS=MjAyMDAzMjEwNDQwMzlfNDc3) | 0.0007332592542007577 | 0.03111717347600229 |
| [UNC93B1 deficiency - HSE](http://www.reactome.org/PathwayBrowser/#/R-HSA-5602415&DTAB=AN&ANALYSIS=MjAyMDAzMjEwNDQwMzlfNDc3) | 0.0007332592542007577 | 0.03111717347600229 |
| [UCH proteinases](http://www.reactome.org/PathwayBrowser/#/R-HSA-5689603&DTAB=AN&ANALYSIS=MjAyMDAzMjEwNDQwMzlfNDc3) | 0.0007589554506342022 | 0.03111717347600229 |
| [Nef mediated downregulation of MHC class I complex cell surface expression](http://www.reactome.org/PathwayBrowser/#/R-HSA-164940&DTAB=AN&ANALYSIS=MjAyMDAzMjEwNDQwMzlfNDc3) | 0.0013748418863652745 | 0.053618833568245705 |
| [mRNA Editing](http://www.reactome.org/PathwayBrowser/#/R-HSA-75072&DTAB=AN&ANALYSIS=MjAyMDAzMjEwNDQwMzlfNDc3) | 0.0017596398496316779 | 0.0633470345867404 |
| [mRNA Editing: A to I Conversion](http://www.reactome.org/PathwayBrowser/#/R-HSA-75064&DTAB=AN&ANALYSIS=MjAyMDAzMjEwNDQwMzlfNDc3) | 0.002858899412564564 | 0.09434368061463061 |
| [C6 deamination of adenosine](http://www.reactome.org/PathwayBrowser/#/R-HSA-75102&DTAB=AN&ANALYSIS=MjAyMDAzMjEwNDQwMzlfNDc3) | 0.002858899412564564 | 0.09434368061463061 |
| [Metalloprotease DUBs](http://www.reactome.org/PathwayBrowser/#/R-HSA-5689901&DTAB=AN&ANALYSIS=MjAyMDAzMjEwNDQwMzlfNDc3) | 0.0037503329544381625 | 0.10925191811698953 |
| [Antigen processing: Ubiquitination & Proteasome degradation](http://www.reactome.org/PathwayBrowser/#/R-HSA-983168&DTAB=AN&ANALYSIS=MjAyMDAzMjEwNDQwMzlfNDc3) | 0.0038640552673664397 | 0.10925191811698953 |
| [DAP12 interactions](http://www.reactome.org/PathwayBrowser/#/R-HSA-2172127&DTAB=AN&ANALYSIS=MjAyMDAzMjEwNDQwMzlfNDc3) | 0.004046367337666279 | 0.10925191811698953 |
| [RMTs methylate histone arginines](http://www.reactome.org/PathwayBrowser/#/R-HSA-3214858&DTAB=AN&ANALYSIS=MjAyMDAzMjEwNDQwMzlfNDc3) | 0.004046367337666279 | 0.10925191811698953 |
| [Cytosolic sensors of pathogen-associated DNA](http://www.reactome.org/PathwayBrowser/#/R-HSA-1834949&DTAB=AN&ANALYSIS=MjAyMDAzMjEwNDQwMzlfNDc3) | 0.004271400865153652 | 0.11105642249399494 |
| [Diseases of Immune System](http://www.reactome.org/PathwayBrowser/#/R-HSA-5260271&DTAB=AN&ANALYSIS=MjAyMDAzMjEwNDQwMzlfNDc3) | 0.004638186457844418 | 0.11131647498826602 |
| [Diseases associated with the TLR signaling cascade](http://www.reactome.org/PathwayBrowser/#/R-HSA-5602358&DTAB=AN&ANALYSIS=MjAyMDAzMjEwNDQwMzlfNDc3) | 0.004638186457844418 | 0.11131647498826602 |

**Table S2:**

**2-1: List of genes that are opposite to ACE2 that are reduced in acute infection and elevated in convalescent SARS-CoV infection [n = 26]**

| ABCC5 | ABLIM1 | AHNAK | C17orf89 | CDCA7L | CEP68 | EIF3L | EIF4B |
| --- | --- | --- | --- | --- | --- | --- | --- |
| EPHA4 | FAM172A | FITM2 | GDF11 | GLS | GSK3B | KLHL36 | NDUFA10 |
| NRBP2 | OIP5-AS1 | PCBD2 | RPL15 | RPL27A | RPL3 | SLC16A7 | SLC35E2 |
| VIPR1 | VPS13A |  |  |  |  |  |  |

**2-2: Reactome analysis for genes opposite to ACE2 that are reduced in acute infection and elevated in convalescent SARS-CoV infection [n = 26]**

| Name | pValue | FDR |
| --- | --- | --- |
| [GTP hydrolysis and joining of the 60S ribosomal subunit](http://www.reactome.org/PathwayBrowser/#/R-HSA-72706&DTAB=AN&ANALYSIS=MjAyMDAzMjIxNTQzNDJfODgx) | 3.514097013379569e-9 | 1.9431648823342584e-7 |
| [L13a-mediated translational silencing of Ceruloplasmin expression](http://www.reactome.org/PathwayBrowser/#/R-HSA-156827&DTAB=AN&ANALYSIS=MjAyMDAzMjIxNTQzNDJfODgx) | 3.514097013379569e-9 | 1.9431648823342584e-7 |
| [Eukaryotic Translation Initiation](http://www.reactome.org/PathwayBrowser/#/R-HSA-72613&DTAB=AN&ANALYSIS=MjAyMDAzMjIxNTQzNDJfODgx) | 6.072390257294558e-9 | 1.9431648823342584e-7 |
| [Cap-dependent Translation Initiation](http://www.reactome.org/PathwayBrowser/#/R-HSA-72737&DTAB=AN&ANALYSIS=MjAyMDAzMjIxNTQzNDJfODgx) | 6.072390257294558e-9 | 1.9431648823342584e-7 |
| [Formation of a pool of free 40S subunits](http://www.reactome.org/PathwayBrowser/#/R-HSA-72689&DTAB=AN&ANALYSIS=MjAyMDAzMjIxNTQzNDJfODgx) | 6.240078331831711e-8 | 0.0000015600195829579278 |
| [Peptide chain elongation](http://www.reactome.org/PathwayBrowser/#/R-HSA-156902&DTAB=AN&ANALYSIS=MjAyMDAzMjIxNTQzNDJfODgx) | 0.0000013850692115457974 | 0.00002829669938364532 |
| [Nonsense Mediated Decay (NMD) independent of the Exon Junction Complex (EJC)](http://www.reactome.org/PathwayBrowser/#/R-HSA-975956&DTAB=AN&ANALYSIS=MjAyMDAzMjIxNTQzNDJfODgx) | 0.0000016858595985880243 | 0.00002829669938364532 |
| [Eukaryotic Translation Elongation](http://www.reactome.org/PathwayBrowser/#/R-HSA-156842&DTAB=AN&ANALYSIS=MjAyMDAzMjIxNTQzNDJfODgx) | 0.0000017685437114778324 | 0.00002829669938364532 |
| [Eukaryotic Translation Termination](http://www.reactome.org/PathwayBrowser/#/R-HSA-72764&DTAB=AN&ANALYSIS=MjAyMDAzMjIxNTQzNDJfODgx) | 0.000002131796673188191 | 0.000029845153424634674 |
| [Selenocysteine synthesis](http://www.reactome.org/PathwayBrowser/#/R-HSA-2408557&DTAB=AN&ANALYSIS=MjAyMDAzMjIxNTQzNDJfODgx) | 0.0000029067716003083888 | 0.00003164524306820127 |
| [Viral mRNA Translation](http://www.reactome.org/PathwayBrowser/#/R-HSA-192823&DTAB=AN&ANALYSIS=MjAyMDAzMjIxNTQzNDJfODgx) | 0.0000030334945886334452 | 0.00003164524306820127 |
| [Response of EIF2AK4 (GCN2) to amino acid deficiency](http://www.reactome.org/PathwayBrowser/#/R-HSA-9633012&DTAB=AN&ANALYSIS=MjAyMDAzMjIxNTQzNDJfODgx) | 0.0000031645243068201268 | 0.00003164524306820127 |
| [SRP-dependent cotranslational protein targeting to membrane](http://www.reactome.org/PathwayBrowser/#/R-HSA-1799339&DTAB=AN&ANALYSIS=MjAyMDAzMjIxNTQzNDJfODgx) | 0.0000037338339450299074 | 0.000033604505505269167 |
| [Translation](http://www.reactome.org/PathwayBrowser/#/R-HSA-72766&DTAB=AN&ANALYSIS=MjAyMDAzMjIxNTQzNDJfODgx) | 0.0000037640053116572147 | 0.00003387604780491493 |
| [Nonsense-Mediated Decay (NMD)](http://www.reactome.org/PathwayBrowser/#/R-HSA-927802&DTAB=AN&ANALYSIS=MjAyMDAzMjIxNTQzNDJfODgx) | 0.000004555432106423396 | 0.00003644345685138717 |
| [Nonsense Mediated Decay (NMD) enhanced by the Exon Junction Complex (EJC)](http://www.reactome.org/PathwayBrowser/#/R-HSA-975957&DTAB=AN&ANALYSIS=MjAyMDAzMjIxNTQzNDJfODgx) | 0.000004555432106423396 | 0.00003644345685138717 |
| [Axon guidance](http://www.reactome.org/PathwayBrowser/#/R-HSA-422475&DTAB=AN&ANALYSIS=MjAyMDAzMjIxNTQzNDJfODgx) | 0.000013776459824810239 | 0.00009643521877367167 |
| [Nervous system development](http://www.reactome.org/PathwayBrowser/#/R-HSA-9675108&DTAB=AN&ANALYSIS=MjAyMDAzMjIxNTQzNDJfODgx) | 0.000021198763962448908 | 0.00014261250385816382 |
| [Influenza Viral RNA Transcription and Replication](http://www.reactome.org/PathwayBrowser/#/R-HSA-168273&DTAB=AN&ANALYSIS=MjAyMDAzMjIxNTQzNDJfODgx) | 0.000023768750643027303 | 0.00014261250385816382 |
| [Selenoamino acid metabolism](http://www.reactome.org/PathwayBrowser/#/R-HSA-2408522&DTAB=AN&ANALYSIS=MjAyMDAzMjIxNTQzNDJfODgx) | 0.000027900157407834136 | 0.00016740094444700482 |
| [Regulation of expression of SLITs and ROBOs](http://www.reactome.org/PathwayBrowser/#/R-HSA-9010553&DTAB=AN&ANALYSIS=MjAyMDAzMjIxNTQzNDJfODgx) | 0.00002939465523721374 | 0.0001712730921954453 |
| [Major pathway of rRNA processing in the nucleolus and cytosol](http://www.reactome.org/PathwayBrowser/#/R-HSA-6791226&DTAB=AN&ANALYSIS=MjAyMDAzMjIxNTQzNDJfODgx) | 0.00003425461843908906 | 0.0001712730921954453 |
| [Influenza Infection](http://www.reactome.org/PathwayBrowser/#/R-HSA-168255&DTAB=AN&ANALYSIS=MjAyMDAzMjIxNTQzNDJfODgx) | 0.00004371898058419532 | 0.0002185949029209766 |
| [rRNA processing in the nucleus and cytosol](http://www.reactome.org/PathwayBrowser/#/R-HSA-8868773&DTAB=AN&ANALYSIS=MjAyMDAzMjIxNTQzNDJfODgx) | 0.00005265641581997382 | 0.0002632820790998691 |
| [Signaling by ROBO receptors](http://www.reactome.org/PathwayBrowser/#/R-HSA-376176&DTAB=AN&ANALYSIS=MjAyMDAzMjIxNTQzNDJfODgx) | 0.00009552483029040548 | 0.0004641491015906496 |
| [rRNA processing](http://www.reactome.org/PathwayBrowser/#/R-HSA-72312&DTAB=AN&ANALYSIS=MjAyMDAzMjIxNTQzNDJfODgx) | 0.0001160372753976624 | 0.0004641491015906496 |
| [Metabolism of amino acids and derivatives](http://www.reactome.org/PathwayBrowser/#/R-HSA-71291&DTAB=AN&ANALYSIS=MjAyMDAzMjIxNTQzNDJfODgx) | 0.0017249564294193886 | 0.006899825717677555 |
| [Cellular responses to stress](http://www.reactome.org/PathwayBrowser/#/R-HSA-2262752&DTAB=AN&ANALYSIS=MjAyMDAzMjIxNTQzNDJfODgx) | 0.002127603149398083 | 0.008510412597592332 |
| [Developmental Biology](http://www.reactome.org/PathwayBrowser/#/R-HSA-1266738&DTAB=AN&ANALYSIS=MjAyMDAzMjIxNTQzNDJfODgx) | 0.002376902588972607 | 0.009507610355890428 |
| [Cellular responses to external stimuli](http://www.reactome.org/PathwayBrowser/#/R-HSA-8953897&DTAB=AN&ANALYSIS=MjAyMDAzMjIxNTQzNDJfODgx) | 0.0024219649238000907 | 0.009687859695200363 |

**Table S2:**

**3-1: 20 gene severe ViP signature [n = 20]**

| CLIC4 | CARD16 | CARD17 | HIST2H2AA4 | HIST1H2AJ | SQRDL | HIST1H2AD | C21orf91 |
| --- | --- | --- | --- | --- | --- | --- | --- |
| B2M | HIST2H2AB | HRASLS2 | GCA | CCNA1 | CASP1 | GCH1 | TRIM25 |
| TMEM92 | IFI27L1 | LOX | ELOVL7 |  |  |  |  |

**3-2: Reactome analysis for severe ViP signature genes [n = 20]**

| Name | pValue | FDR |
| --- | --- | --- |
| [RMTs methylate histone arginines](http://www.reactome.org/PathwayBrowser/) | 2.92E-09 | 6.66E-07 |
| [Metalloprotease DUBs](http://www.reactome.org/PathwayBrowser/) | 1.37E-08 | 1.567E-06 |
| [HDACs deacetylate histones](http://www.reactome.org/PathwayBrowser/) | 3.86E-07 | 2.934E-05 |
| [DNA Damage/Telomere Stress Induced Senescence](http://www.reactome.org/PathwayBrowser/) | 6.93E-07 | 3.947E-05 |
| [Packaging Of Telomere Ends](http://www.reactome.org/PathwayBrowser/) | 1.32E-06 | 4.987E-05 |
| [RNA Polymerase I Promoter Opening](http://www.reactome.org/PathwayBrowser/) | 1.32E-06 | 4.987E-05 |
| [DNA methylation](http://www.reactome.org/PathwayBrowser/) | 1.654E-06 | 4.987E-05 |
| [Amyloid fiber formation](http://www.reactome.org/PathwayBrowser/) | 1.969E-06 | 4.987E-05 |
| [Senescence-Associated Secretory Phenotype (SASP)](http://www.reactome.org/PathwayBrowser/) | 2.196E-06 | 4.987E-05 |
| [Recognition and association of DNA glycosylase with site containing an affected purine](http://www.reactome.org/PathwayBrowser/) | 2.267E-06 | 4.987E-05 |
| [Recognition and association of DNA glycosylase with site containing an affected pyrimidine](http://www.reactome.org/PathwayBrowser/) | 3.034E-06 | 5.638E-05 |
| [UCH proteinases](http://www.reactome.org/PathwayBrowser/) | 3.317E-06 | 5.638E-05 |
| [PRC2 methylates histones and DNA](http://www.reactome.org/PathwayBrowser/) | 3.642E-06 | 5.638E-05 |
| [SIRT1 negatively regulates rRNA expression](http://www.reactome.org/PathwayBrowser/) | 3.978E-06 | 5.638E-05 |
| [Cleavage of the damaged purine](http://www.reactome.org/PathwayBrowser/) | 3.978E-06 | 5.638E-05 |
| [Deubiquitination](http://www.reactome.org/PathwayBrowser/) | 4.068E-06 | 5.638E-05 |
| [Depurination](http://www.reactome.org/PathwayBrowser/) | 4.337E-06 | 5.638E-05 |
| [ERCC6 (CSB) and EHMT2 (G9a) positively regulate rRNA expression](http://www.reactome.org/PathwayBrowser/) | 5.124E-06 | 6.149E-05 |
| [Activated PKN1 stimulates transcription of AR (androgen receptor) regulated genes KLK2 and KLK3](http://www.reactome.org/PathwayBrowser/) | 5.556E-06 | 6.372E-05 |
| [HATs acetylate histones](http://www.reactome.org/PathwayBrowser/) | 5.793E-06 | 6.372E-05 |
| [Cleavage of the damaged pyrimidine](http://www.reactome.org/PathwayBrowser/) | 6.498E-06 | 6.498E-05 |
| [Depyrimidination](http://www.reactome.org/PathwayBrowser/) | 6.498E-06 | 6.498E-05 |
| [Ub-specific processing proteases](http://www.reactome.org/PathwayBrowser/) | 7.814E-06 | 6.986E-05 |
| [Nucleosome assembly](http://www.reactome.org/PathwayBrowser/) | 8.127E-06 | 6.986E-05 |
| [Deposition of new CENPA-containing nucleosomes at the centromere](http://www.reactome.org/PathwayBrowser/) | 8.127E-06 | 6.986E-05 |
| [Condensation of Prophase Chromosomes](http://www.reactome.org/PathwayBrowser/) | 8.732E-06 | 6.986E-05 |
| [Meiotic recombination](http://www.reactome.org/PathwayBrowser/) | 1.075E-05 | 8.596E-05 |
| [B-WICH complex positively regulates rRNA expression](http://www.reactome.org/PathwayBrowser/) | 1.394E-05 | 9.464E-05 |
| [Meiotic synapsis](http://www.reactome.org/PathwayBrowser/) | 1.394E-05 | 9.464E-05 |
| [Base-Excision Repair, AP Site Formation](http://www.reactome.org/PathwayBrowser/) | 1.394E-05 | 9.464E-05 |
