# Supplementary material for: AI-guided discovery of the invariant host response to viral pandemics
Source: eBioMedicine. 2021 Jun 11;68:103390. doi: 10.1016/j.ebiom.2021.103390 (PMC8193764; doi:10.1016/j.ebiom.2021.103390)
Supplement: Supplementary file 3 [file mmc3.docx]

**Table S3. Virus Infection Datasets Used in this Study**

| Virus | Type (RNA/DNA) | Portal of Entry (CME-dependent or independent) | GSE ID | In vitro/in vivo | Cell type | ROC AUC |
| --- | --- | --- | --- | --- | --- | --- |
| HIV | ss RNA (+) | CME-dependent | GSE125817 | in vitro | human monocyte derived dendritic cells (moDCs) | 1 |
| Zika | ss RNA (+) | CME-dependent | GSE146423 | in vitro | A549 cells (Adenocarcinomic human alveolar basal epithelial cells) | 1 |
| WNV | ssRNA (+) | CME-dependent | GSE138841 | in vitro | A549 cells (Adenocarcinomic human alveolar basal epithelial cells) | 1 |
| WNV | ssRNA (+) | CME-dependent | GSE136342 | in vitro | Human monocyte derived DCs moDCs | 1 |
| CoV (MERS) | ss RNA (+) | CME-dependent | GSE56677 | In vitro | Calu-3 2B4 cells; Human Coronavirus EMC 2012 (HCoV-EMC) | 1 |
| CoV (HCoV-EMC) | ss RNA (+) | CME-dependent | GSE45042 | In vitro | Calu-3 2B4 cells were infected with Human Coronavirus EMC 2012 (HCoV-EMC) | 1 |
| CoV | ss RNA (+) | CME-dependent | GSE17400 | In vitro | Calu3 cells with SARS-CoV1 and Dhori virus (DHOV), a member of the Orthomyxoviridae family within the Thogotovirus genus | 1 |
| CoV | ss RNA (+) | CME-dependent | GSE30589 | In vitro | Vero E6 cells SARS-CoV-1 | 1 |
| H1N1 | ss RNA (-) | CME-dependent | GSE47963 | In vitro | HAE cultures with H1N1 Inf virus | 1 |
| Inf A/B | ss RNA (-) | CME-dependent | GSE68310 | in vivo (human) | Peripheral blood cells | 0.97 |
| CoV (SARS-CoV-1) | ss RNA (+) | CME-dependent | GSE33267 | In vitro | Calu-3 cells were infected with either icSARS CoV or the icSARS deltaORF6 | 0.97 |
| EnV HHV-6 | ds DNA | CME-independent | GSE40396 | in vivo (human) | Peripheral blood cells | 0.95 |
| CoV (SARS-CoV-1) | ss RNA (+) | CME-dependent | GSE37827 | In vitro | Calu-3 cells were infected with either icSARS CoV or the cSARS Bat SRBD | 0.94 |
| HIV | ss RNA (+) | CME-dependent | GSE140713 | in vivo (human) | PBMCs | 0.93 |
| HCV | ss RNA (+) | CME-dependent | GSE40184 | in vivo (human) | PBMCs | 0.93 |
| HSV | ds DNA | CME-independent | GSE46042 | in vitro | Pluripotent stem cell derived neuronallineages | 0.89 |
| Ebola | ss RNA (-) | CME-dependent (and Macropinocytosis) | GSE130629 | in vivo (mouse) | Liver and spleen | 0.88 |
| HAV | ss RNA (+) | CME-dependent | GSE40396 | in vivo (human) | Peripheral blood cells | 0.87 |
| CMV | ds DNA | CME-independent | GSE81246 | in vivo (human) | PBMCs | 0.86 |
| SARS-CoV-2 | ss RNA (+) | CME-dependent | GSE147507 | In vitro | Primary human lung epithelium (NHBE) and transformed lung alveolar (A549) cells were mock treated or infected with SARS-CoV-2 (USA-WA1/2020) | 0.86 |
| HHV6 | ds DNA | CME-independent | GSE40396 | in vivo (human) | Peripheral blood cells | 0.85 |
| HEV | ss RNA (+) | CME-dependent | GSE36539 | in vivo (human) | Peripheral blood cells | 0.85 |
| VZV | ds DNA | CME-independent | GSE136586 | in vitro | Primary keratinocytes | 0.81 |
| SARS-CoV1 | ss RNA (+) | CME-dependent | GSE47963 | In vitro | HAE cultures with SARS-CoV1 | 0.81 |
| VZV | ds DNA | CME-independent | GSE54385 | in vitro | human dermal fibroblasts and neuron | 0.75 |
| HBV | ds DNA | CME-dependent | GSE135501 | in vivo (human) | Peripheral blood cells | 0.71 |
| AdV | ss DNA | CME-dependent | GSE40396 | in vivo (human) |  | 0.71 |
| HRV | ss RNA (+) | CME-dependent | GSE40396 | in vivo (human) |  | 0.7 |
| CMV | ds DNA | CME-independent | GSE99454 | in vitro | MRC-5 and ARPE-19 cells | 0.65 |
| EBV | ds DNA | CME-dependent | GSE135644 | in vitro | AGS and AGS-EBV cells | 0.64 |
| Measles | ss RNA (-) | CME-independent (Caveolin-dependent) | GSE5808 | in vivo | PBMC | 0.53 |
| HPV | ds DNA | CME-independent | GSE137965 | in vitro | Primary keratinocytes | 0.51 |
