# Supplementary material for: AI-guided discovery of the invariant host response to viral pandemics
Source: eBioMedicine. 2021 Jun 11;68:103390. doi: 10.1016/j.ebiom.2021.103390 (PMC8193764; doi:10.1016/j.ebiom.2021.103390)
Supplement: Supplementary file 4 [file mmc4.docx]

**Table S4. Table of 20 genes that define “severity” within the 166-gene ViP signature**

| Gene Name | Host responses/infection | Targeted pathway | Drug/biologics-FDA approved |
| --- | --- | --- | --- |
| CLIC4 | Chloride intracellular channels 4 is required for the replication of Chikungunya virus (PMID: 31483794) | - | Ribavirin PMID: 31483794 |
| CARD16 | CARD 16 increases after the influenza A virus (PMID: 29299535) | Therapeutics on NLRP3  NLRP3 inhibitor— | Canakinumab  Anakinra  rilonacept |
| CARD17 | CARD 17 increases after Chikungunya virus (CHIKV) infection (PMID: 31211814) & after the influenza A virus (PMID: 29299535) | Therapeutics on NLRP3  NLRP3 inhibitor— | Canakinumab  Anakinra  rilonacept |
| HIST2H2AA4 | Histone family | pan-histone deacetylase (HDAC) inhibitor SAHA | SAHA |
| HIST1H2AJ | Histone family | pan-histone deacetylase (HDAC) inhibitor SAHA | SAHA |
| SQRDL | Sulfide quinone reductase |  |  |
| HIST1H2AD | Histone family | pan-histone deacetylase (HDAC) inhibitor SAHA | SAHA |
| C21orf91 | Cold Sore Susceptibility Gene 1, influences pathogenesis of herpes virus (PMID: 27081513) | - |  |
| B2M | b2 microglobulin mRNA levels are transiently increased after infection with Neurovirulent Sindbis Virus, suggesting that transcription of these mRNAs are coordinately  regulated in neurons. (PMCID: PMC112110 |  |  |
| HIST2H2AB | Histone family | pan-histone deacetylase (HDAC) inhibitor SAHA | SAHA |
| HRASLS2 | Phospholipase A And Acyltransferase 2 |  |  |
| GCA | Grancalcin/GCA heterodimerization of TLR9 is important for TLR9-mediated downstream signaling during fine tune processes against viral infection (PMID: 26648480) |  |  |
| CCNA1 | Cyclin A1 |  |  |
| CASP1 | Caspase 1 |  |  |
| GCH1 | GTP cyclohydrolase;  Neopterin is a biomarker for viral infection  It is produced as a by-product in tetrahydrobiopterin (BH4) de novo synthesis and mirrors the activity of  the rate limiting enzyme in the BH4 synthesis cascade |  |  |
| TRIM25 | tripartite motif containing 25 (TRIM25) is an E3 ubiquitin ligase and activates RIG1. It is involved in innate immune responses regulates intracellular signaling and/or RNA virus replication. PMID: 29018447 |  |  |
| TMEM92 | Transmembrane Protein 92 |  |  |
| IFI27L1 | Interferon Alpha Inducible Protein 27 Like 1 |  |  |
| LOX | Lipoxygenase increased in lung cells during RSV infection. It also gives higher CCL3 and CCL5 (PMID: 28579398) | Maraviroc prevent the function of CCR5 receptor and it will block CCL5 binding (PMID: 22637726) | Maraviroc |
| ELOVL7 | host fatty acid elongase 7; required for efficient virus release and virion infectivity (PMID: 25732827) |  |  |
