# Supplementary material for: AI-guided discovery of the invariant host response to viral pandemics
Source: eBioMedicine. 2021 Jun 11;68:103390. doi: 10.1016/j.ebiom.2021.103390 (PMC8193764; doi:10.1016/j.ebiom.2021.103390)
Supplement: Supplementary file 5 [file mmc5.docx]

**Table S5. Demographics of the UCSD COVID-19 cohort participants for plasma.**

| UCSD ID | Acute/ Convalescent | Days post symptom onset | Age | Gender | Peak Disease Severity |
| --- | --- | --- | --- | --- | --- |
| CoV3 | Acute | 37 | 59 | M | Critical |
| CoV23 | Acute | 18 | 72 | F | Moderate |
| CoV36 | Acute | 17 | 43 | F | Critical |
| CoV37 | Acute | 4 | 25 | F | Mild |
| CoV38 | Acute | 4 | 32 | M | Mild |
| CoV41 | Acute | 7 | 83 | M | Mild |
| CoV40 | Acute | 10 | 83 | M | Severe to Critical (NRB in ICU but never intubated) |
| CoV42 | Acute | 6 | 58 | M | Mild to Moderate |
| CoV64 | Acute | 13 | 62 | M | Moderate |
| CoV65 | Acute | 28 | 42 | M | Critical |
| CoV66 | Acute | 6 | 53 | F | Moderate |
| Cov77 | Acute | 9 | 81 | M | Mod-Severe |
| Cov78 | Acute | 10 | 42 | M | Mod-Severe |
| CoV81 | Acute | 22 | 37 | F | Critical |
| CoV82 | Acute | 8 | 53 | M | Critical |
| CoV92 | Acute | 10 | 84 | M | Critical |
| CoV93 | Acute | 4 | 41 | M | Critical |
| CoV97 | Acute | 13 | 65 | M | Critical |
| CoV98 | Acute | 5 | 86 | M | Critical |
| CoV99 | Acute | 22 | 77 | F | Critical |
| CoV14 | Convalescent | 24 | 32 | M | Mild |
| CoV21 | Convalescent | 27 | 42 | M | Mild |
| CoV22 | Convalescent | 34 | 51 | M | Mild |
| CoV25 | Convalescent | 21 | 40 | F | Mild |
| CoV26 | Convalescent | 30 | 43 | F | Mild |
| CoV29 | Convalescent | 30 | 55 | F | Mild |
| CoV30 | Convalescent | 27 | 40 | M | Mild to Moderate |
| CoV31 | Convalescent | 33 | 64 | M | Mild |
| Cov7 | Convalescent | 25 | 44 | F | mild |
| Cov8 | Convalescent | 22 | 45 | M | mild |
| Cov9 | Convalescent | 25 | 43 | F | mild |
| Cov12 | Convalescent | 25 | 39 | F | mild |
| Cov18 | Convalescent | 23 | 48 | F | mild |
| Cov62 | Convalescent | 50 | 55 | M | mild |
| Cov63 | Convalescent | 39 | 68 | M | mild to moderate |
| Cov64 | Convalescent | 56 | 44 | F | mild |
| Cov201 | Acute | 12 | 73 | M | moderate |
| Cov204 | Acute | 13 | 54 | M | moderate |
| Cov205 | Acute | 9 | 37 | M | moderate |
| Cov206 | Acute | 10 | 77 | F | moderate |
| Cov207 | Acute | 8 | 49 | M | moderate |
| Cov210 | Acute | 5 | 48 | M | moderate |
| Cov211 | Acute | 8 | 39 | F | moderate |
| Cov202 | Acute | 10 | 37 | M | severe |
| Cov106 | Convalescent | 74 | 59 | M | Moderate |
| Cov103 | Convalescent | 79 | 50 | M | mild to moderate |
| Cov208A | Acute | 7 | 84 | M | Fatal |
| Cov213A | Acute | NA | 37 | M | Asymptomatic |
| Cov215 | Acute | 9 | 68 | M | Severe |
| Cov218 | Acute | 5 | 53 | M | Severe |
| CoV220 | Acute | N/A | 55 | M | Asymptomatic |
| CoV221 | Acute | 5 | 68 | M | moderate to severe |
| CoV222 | Acute | 5 | 68 | M | moderate |
| CoV223 | Acute | 7 | 57 | M | moderate to severe |
