# Supplementary material for: AI-guided discovery of the invariant host response to viral pandemics
Source: eBioMedicine. 2021 Jun 11;68:103390. doi: 10.1016/j.ebiom.2021.103390 (PMC8193764; doi:10.1016/j.ebiom.2021.103390)
Supplement: Supplementary file 6 [file mmc6.docx]

**Table S6. Demographics of the UCSD COVID-19 cohort participants for lung tissue.**

| Sample Name | Age | Gender | Days spent hospitalized COVID + until expired | Smoking history | Drugs administered during hospitalization | Subsequent infection? | Reason for surgery (NON SARS-CoV-2) | Histology (NON SARS-CoV-2) |
| --- | --- | --- | --- | --- | --- | --- | --- | --- |
| Autopsy 2 | 64 | Male | 35 days | Smoked 1.5-2 Packs per day (PPD) until 1988 when they quit. Smoked cannabis daily | Lasix (furosemide), Amiodarone HCL, stress dose steroids, vancomycin/zosyn, IV ceftriaxone, norepinephrine and vasopressin, digoxin, vecuronium | MSSA from being intubated |  |  |
| Autopsy 3 | NA | NA | NA | NA | NA | NA |  |  |
| Autopsy 4 | 90 | Female | 3 | NA | vancomycin/zosyn, low dose of norepinephrine | none |  |  |
| Autopsy 5 | 57 | Male | 28 | NA | Cefepime, Hydroxychloroquine, vancomycin, Norepinephrine, vasopressin, esmolol, Unasyn, bivalirudin, pressors (levo, vaso, phenylephrine), linezolid ,Tocilizumab v. Placebo (information was not given what group he belonged to) | E.feacalis and klebsillea PNA from tracheal aspirate |  |  |
| JA 5 (NON SARS-CoV-2) | 46 | Female | NA | Non-smoker | NA | NA | Left Lower Lobe Nodule | Invasive Adenocarcinoma |
| JA 6 (NON SARS-CoV-2) | 70 | Male | NA | Non-smoker | NA | NA | Left Lower Lobe Nodule | Granulomatous-like inflammation and scar |
