# Supplementary material for: AI-guided discovery of the invariant host response to viral pandemics
Source: eBioMedicine. 2021 Jun 11;68:103390. doi: 10.1016/j.ebiom.2021.103390 (PMC8193764; doi:10.1016/j.ebiom.2021.103390)
Supplement: Supplementary file 7 [file mmc7.pdf]

## SUPPLEMENTARY ONLINE MATERIALS

**Title: AI-guided discovery of the invariant host response to viral pandemics**

**Authors:** Debashis Sahoo[1-3†*], Gajanan D. Katkar[4*], Soni Khandelwal[1], Mahdi Behroozikhah[2], Amanraj Claire[4], Vanessa Castillo[4], Courtney Tindle[4], MacKenzie Fuller[4], Sahar Taheri[2], Thomas F. Rogers[5-6], Nathan Beutler[5], Sydney I. Ramirez[10, 11], Stephen A. Rawlings[11], Victor Pretorius[14], Davey M. Smith[11], Dennis R. Burton[5, 7-8], Laura E. Crotty Alexander[9], Jason Duran[15], Shane Crotty[10, 11], Jennifer M. Dan[10, 11], Soumita Das[11†] and Pradipta Ghosh[4,13†]

## Affiliations:

*[1]Department of Pediatrics, University of California San Diego.*
*[2]Department of Computer Science and Engineering, Jacobs School of Engineering, University of California San Diego.*
*[3]Moores Cancer Center, University of California San Diego.*
*[4]Department of Cellular and Molecular Medicine, University of California San Diego.*
*[5]Department of Immunology and Microbiology, The Scripps Research Institute, La Jolla, CA 92037, USA.*
*[6]Division of Infectious Diseases, Department of Medicine, University of California, San Diego, La Jolla, CA 92037, USA.*
*[7]IAVI Neutralizing Antibody Center, The Scripps Research Institute, La Jolla, CA 92037, USA.*
*[8]Consortium for HIV/AIDS Vaccine Development (CHAVD), The Scripps Research Institute, La Jolla, CA 92037, USA.*
*[9]Pulmonary Critical Care Section, Veterans Affairs (VA) San Diego Healthcare System, La Jolla, California; Division of Pulmonary, Critical Care and Sleep Medicine, Department of Medicine, University of California San Diego (UCSD), La Jolla, California*
*[10]Center for Infectious Disease and Vaccine Research, La Jolla Institute for Immunology (LJI), La Jolla, CA, USA.*
*[11]Department of Medicine, Division of Infectious Diseases and Global Public Health, University of California, San Diego (UCSD), La Jolla, CA, USA.*
*[12]Department of Pathology, University of California San Diego.*
*[13]Medicine, University of California San Diego.*
*[14]Department of Surgery, University of California San Diego.*
*[15]Division of Cardiology, Department of Internal Medicine, UC San Diego Medical Center, La Jolla 92037*

*Equal contribution
† Co-Corresponding

## Corresponding authors:

**Debashis Sahoo, Ph.D.;** Assistant Professor, Department of Pediatrics, University of California San Diego; 9500 Gilman Drive, MC 0703, Leichtag Building 132; La Jolla, CA 92093-0831. **Phone:** 858-246-1803: **Fax:** 858-246-0019: **Email:** dsahoo@ucsd.edu

**Soumita Das, Ph.D.;** Associate Professor, Department of Pathology, University of California San Diego; 9500 Gilman Drive, George E. Palade Bldg, Rm 256; La Jolla, CA 92093. **Phone**: 858-246-2062: **Email:** sodas@ucsd.edu

**Pradipta Ghosh, M.D.;** Professor, Departments of Medicine and Cell and Molecular Medicine, University of California San Diego; 9500 Gilman Drive (MC 0651), George E. Palade Bldg, Rm 232; La Jolla, CA 92093. **Phone**: 858-822-7633: **Email:** prghosh@ucsd.edu

**SUPPLEMENTARY MATERIALS: Includes**

- Supplementary Text- **n/a**
- Figures **S1-S4**
- Tables **S1-6**
- External Databases **- None**
- References ***(1-20)***

## SUPPLEMENTARY FIGURES AND LEGENDS

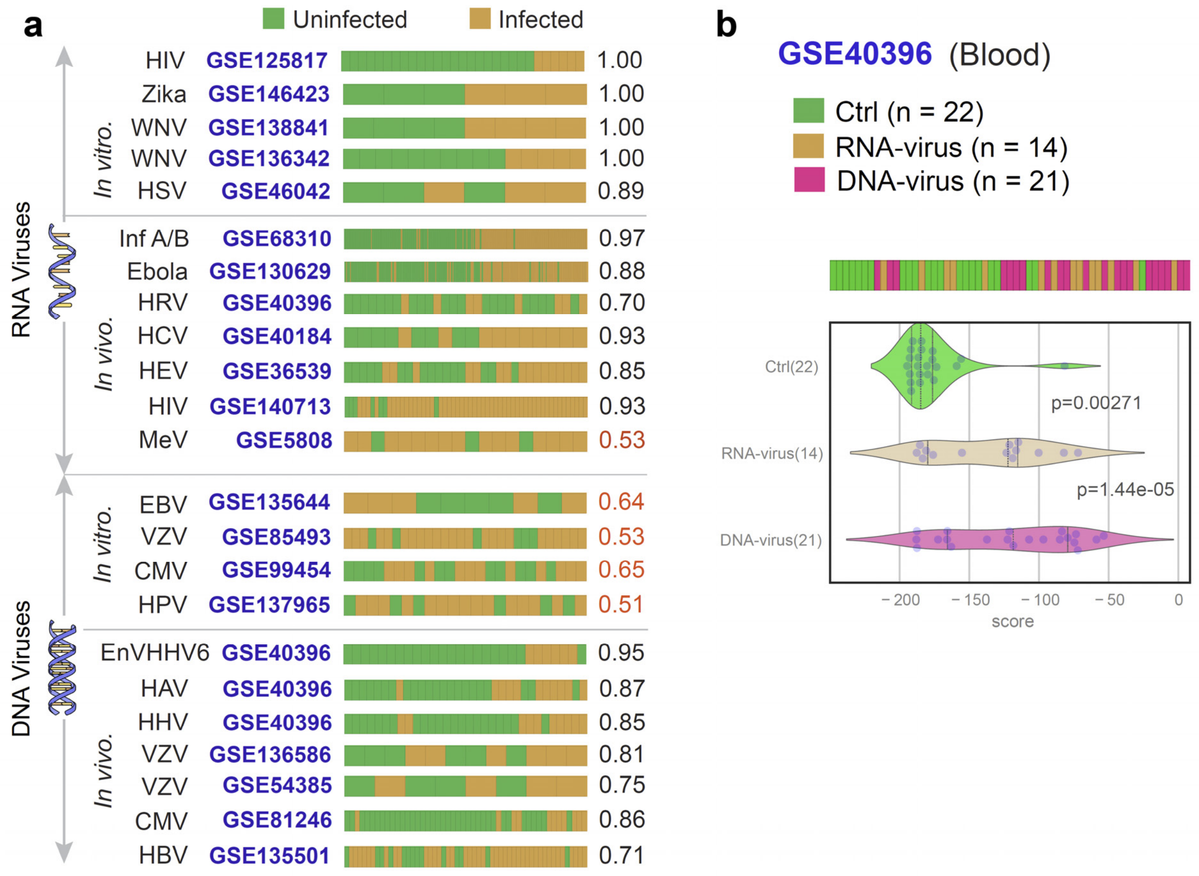


**Figure S1. The ViP signature distinguishes infected from uninfected samples across multiple RNA and DNA viruses. (a)** Bar plots showing the accuracy of the 166 gene ViP signature to classify infected vs. uninfected samples. Samples are categorized based on the nature of genetic material in the viruses (RNA *vs*. DNA) and whether the dataset was generated by assessing infections in *in vitro* cell-based models or in infected humans (*in vivo*). ROC-AUC values of infected samples classifications are shown on the right side of each bar plot. See also **Table S3**, which classifies these viruses based on their route of entry into cells, i.e., clathrin-dependent *vs*. independent endocytosis. ROC-AUC values of infected samples classifications are shown on the right side of each bar plot. **(b)** Bar (top) and violin (bottom) plots show that the ViP signature is equally effective in distinguishing RNA (R) and DNA (D) virus infections from uninfected controls (Ctrl) *in vivo.*

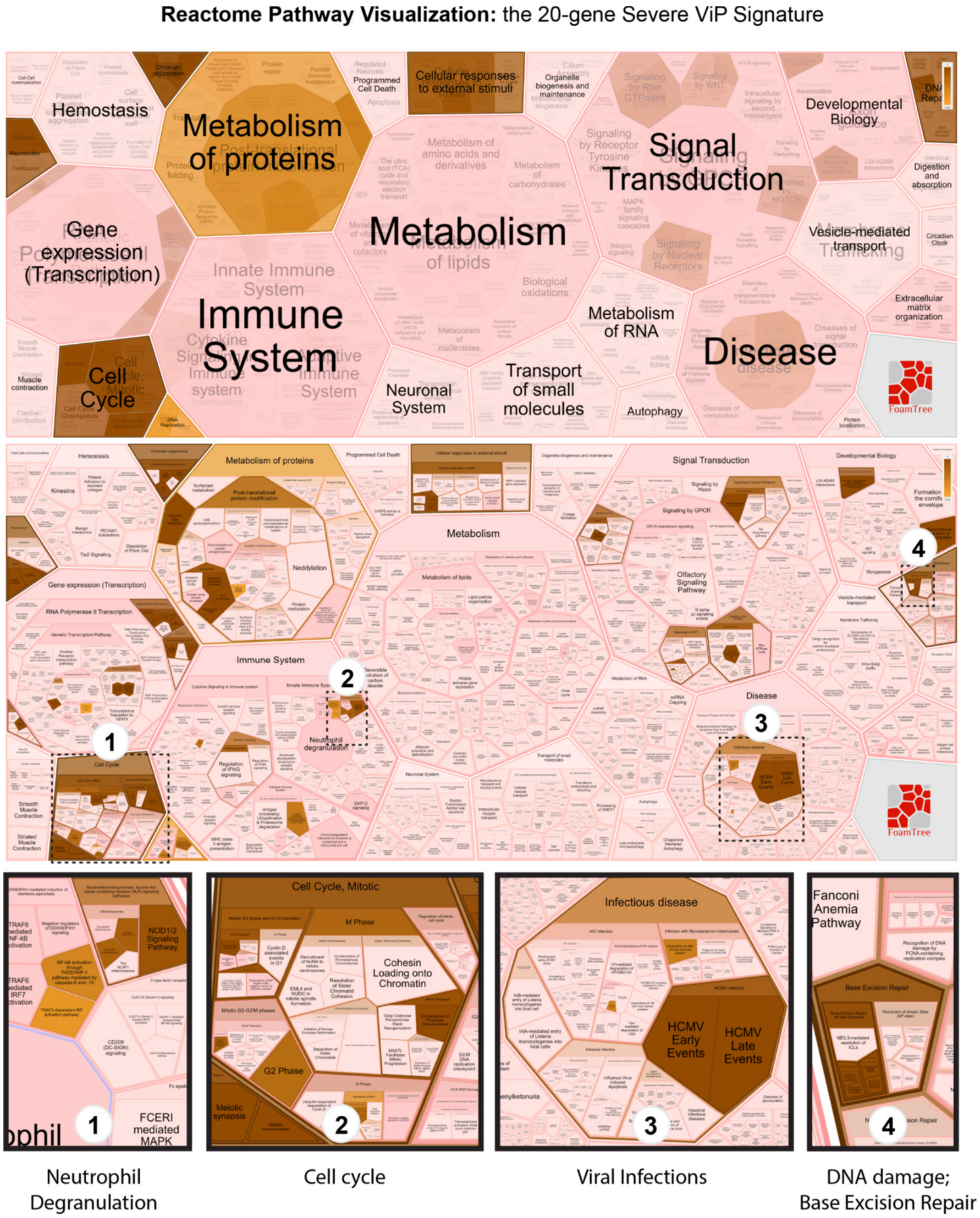


**Figure S2. Reacfoam analysis of 20-gene severe ViP signature. (A)** ReacFome pathway analysis of 20 gene severe ViP signature and visualization based on Voronoi tessellation. Cell cycle, Immune System, Disease and DNA Damage components are highlighted.

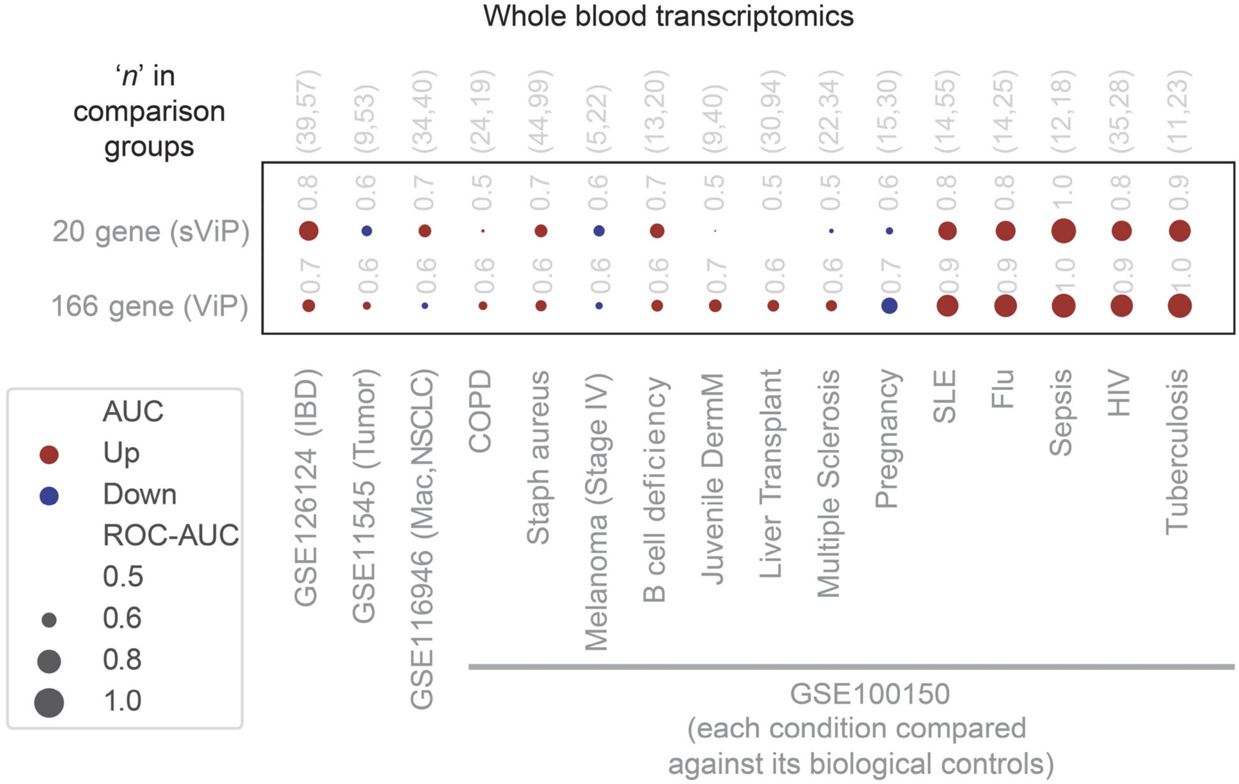


**Figure S3. ViP signatures are specific for diseases of infectious and inflammatory conditions.** Bubble plots showing up- (red) or downregulation (blue) of *ViP* signatures in blood samples from patients with diverse conditions and their corresponding controls. The size of bubble indicates the accuracy of classification (AUC ROC; see key) of controls from diseases samples using the signatures. Each dataset contains its own biological controls, and there are no replicates. IBD, Inflammatory bowel disease; NSCLC, non-small cell lung cancer; COPD, chronic obstructive pulmonary disease; Staph, staphylococcal infection; SLE, systemic lupus erythematosus.

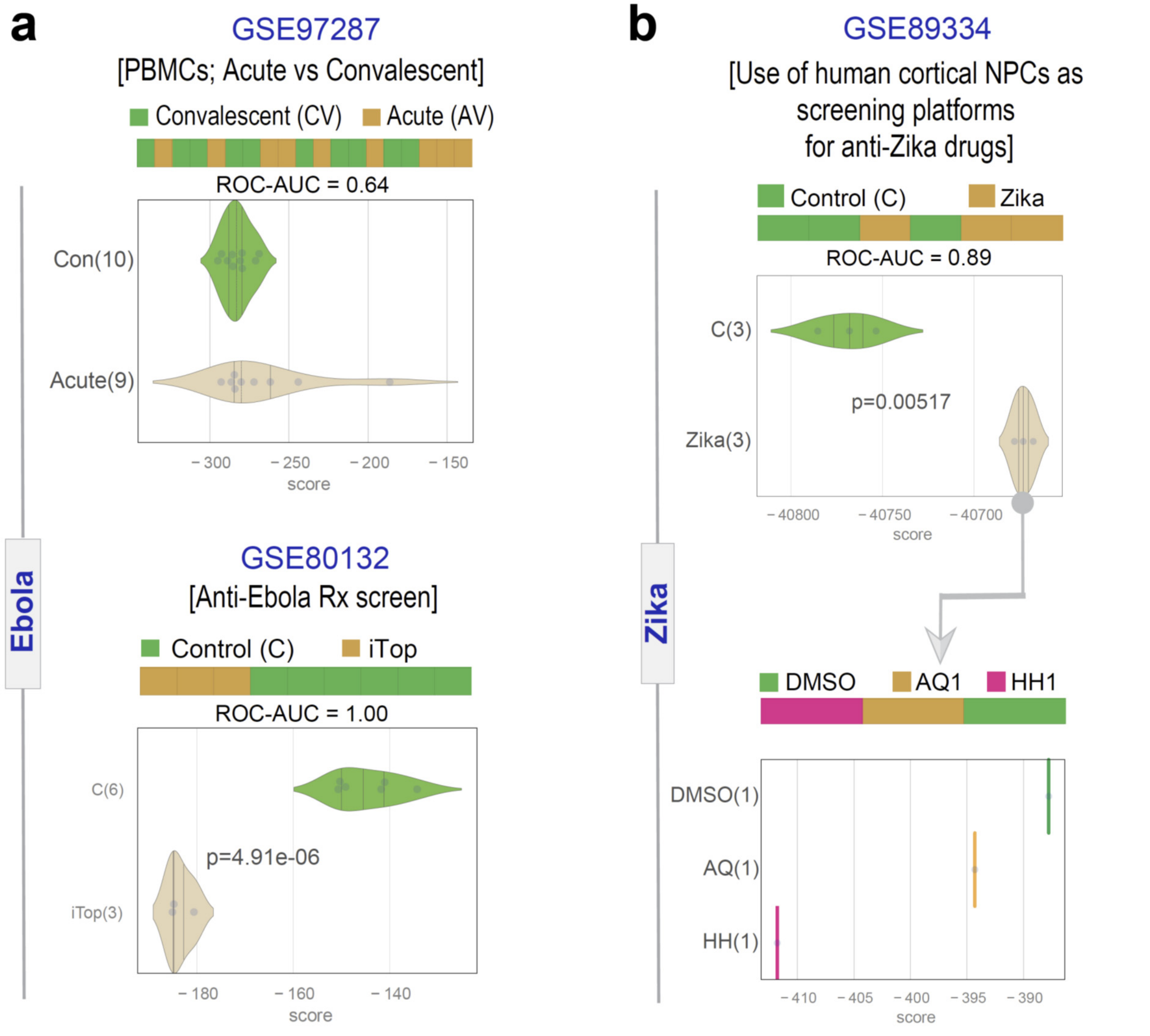


**Figure S4. Validation of ViP signature-guided therapeutic goals.**
**(a)** *Top*: 166-gene ViP signature-based classification of crisis and convalescence in PBMCs from patients with Ebola infection. *Bottom*: The effect of inhibiting Topoisomerase 1 (iTop) in a cultured cell line model infected in vitro with Ebola for the development of anti-Ebola therapeutics.
**(b)** 166-gene ViP signature-based classification of human cortical neural progenitor cells infected *in vitro* with Zika virus (*top*) and the infected cells treated with two investigational drugs (*bottom*; two treatments, AQ1 and HH1) during screening assays.

**SUPPLEMENTARY TABLES INDEX**: (Uploaded separately)

**Table S1:** Catalog of publicly available datasets analyzed in this work

**Table S2**: Gene clusters that constitute the ViP signature and their reactome analyses

**Table S3**: Classification of host response to viral infection using the ViP signature

**Table S4**: Table of 20 genes that define "severity" within the 166-gene ViP signature

**Table S5**. Demographics of the UCSD COVID-19 cohort participants for plasma.

**Table S6**. Demographics of the UCSD COVID-19 cohort participants for lung tissue.

## REFERENCES:


1. Barrett T, Suzek TO, Troup DB, Wilhite SE, Ngau WC, Ledoux P, et al. NCBI GEO: mining millions of expression profiles--database and tools. Nucleic Acids Res. 2005;33(Database issue):D562-6.
2. Barrett T, Wilhite SE, Ledoux P, Evangelista C, Kim IF, Tomashevsky M, et al. NCBI GEO: archive for functional genomics data sets--update. Nucleic Acids Res. 2013;41(Database issue):D991-5.
3. Edgar R, Domrachev M, Lash AE. Gene Expression Omnibus: NCBI gene expression and hybridization array data repository. Nucleic Acids Res. 2002;30(1):207-10.
4. Irizarry RA, Bolstad BM, Collin F, Cope LM, Hobbs B, Speed TP. Summaries of Affymetrix GeneChip probe level data. Nucleic Acids Res. 2003;31(4):e15.
5. Irizarry RA, Hobbs B, Collin F, Beazer-Barclay YD, Antonellis KJ, Scherf U, et al. Exploration, normalization, and summaries of high density oligonucleotide array probe level data. Biostatistics. 2003;4(2):249-64.
6. Li B, Dewey CN. RSEM: accurate transcript quantification from RNA-Seq data with or without a reference genome. BMC Bioinformatics. 2011;12:323.
7. Pachter L. Models for transcript quantification from RNA-Seq. arXiv e-prints [Internet]. 2011 April 01, 2011. Available from: https://ui.adsabs.harvard.edu/\#abs/2011arXiv1104.3889P.
8. Rogers TF, Zhao F, Huang D, Beutler N, Burns A, He WT, et al. Isolation of potent SARS-CoV-2 neutralizing antibodies and protection from disease in a small animal model. Science. 2020;369(6506):956-63.
9. Varghese F, Bukhari AB, Malhotra R, De A. IHC Profiler: an open source plugin for the quantitative evaluation and automated scoring of immunohistochemistry images of human tissue samples. PLoS One. 2014;9(5):e96801.
10. Sahoo D, Dill DL, Tibshirani R, Plevritis SK. Extracting binary signals from microarray time-course data. Nucleic Acids Res. 2007;35(11):3705-12.
11. Sahoo D, Dill DL, Gentles AJ, Tibshirani R, Plevritis SK. Boolean implication networks derived from large scale, whole genome microarray datasets. Genome Biol. 2008;9(10):R157.
12. Sahoo D, Seita J, Bhattacharya D, Inlay MA, Weissman IL, Plevritis SK, et al. MiDReG: a method of mining developmentally regulated genes using Boolean implications. Proc Natl Acad Sci U S A. 2010;107(13):5732-7.
13. Pandey S, Sahoo D. Identification of gene expression logical invariants in Arabidopsis. Plant Direct. 2019;3(3):e00123.
14. Dabydeen SA, Desai A, Sahoo D. Unbiased Boolean analysis of public gene expression data for cell cycle gene identification. Mol Biol Cell. 2019;30(14):1770-9.
15. Jones AC, Anderson D, Galbraith S, Fantino E, Gutierrez Cardenas D, Read JF, et al. Personalized Transcriptomics Reveals Heterogeneous Immunophenotypes in Children with Viral Bronchiolitis. Am J Respir Crit Care Med. 2019;199(12):1537-49.
16. Stuart T, Butler A, Hoffman P, Hafemeister C, Papalexi E, Mauck WM, 3rd, et al. Comprehensive Integration of Single-Cell Data. Cell. 2019;177(7):1888-902 e21.
17. Zhang Z, Luo D, Zhong X, Choi JH, Ma Y, Wang S, et al. SCINA: A Semi-Supervised Subtyping Algorithm of Single Cells and Bulk Samples. Genes (Basel). 2019;10(7).
18. Fabregat A, Jupe S, Matthews L, Sidiropoulos K, Gillespie M, Garapati P, et al. The Reactome Pathway Knowledgebase. Nucleic Acids Res. 2018;46(D1):D649-D55.
